# Atmospheric Reactivity and Environmental Implications of 2‑Propoxyethanol: Kinetic and Mechanistic Insights into Its Oxidation by OH, NO_3_, and Cl

**DOI:** 10.1021/acsenvironau.6c00061

**Published:** 2026-06-18

**Authors:** Sagrario Salgado, Inmaculada Aranda, Pilar Martín, Florentina Villanueva, Elena Molina, Beatriz Cabañas

**Affiliations:** † Departamento de Química Física, Facultad de Ciencias y Tecnologías Químicas, 16733 Universidad de Castilla-La Mancha, Avda. Camilo José Cela s/n, Ciudad Real 13071, Spain; ‡ Instituto de Combustión y Contaminación Atmosférica (ICCA), Universidad de Castilla-La Mancha, Camino de los Moledores s/n, Ciudad Real 13071, Spain; § Departamento de Química Física, Escuela de Ingeniería Industrial y Aeroespacial, Universidad de Castilla-La Mancha, Avenida Carlos III, s/n, Toledo 45003, Spain

**Keywords:** hydroxy ethers, atmospheric chemistry, rate coefficients, reaction products, atmospheric implications

## Abstract

Hydroxyethers (HEs) are widely used oxygenated volatile organic compounds (OVOCs). Among them, 2-propoxyethanol (CH_3_CH_2_CH_2_OCH_2_CH_2_OH, 2-PE) is of particular interest due to its extensive use and the limited mechanistic information available regarding its atmospheric degradation. These characteristics place 2-PE as a compound of emerging environmental interest and motivate a detailed investigation of its reactivity with key atmospheric oxidants. This compound is commonly used as a solvent and emulsifying agent for hydrophobic substances, and it has been proposed as a biodiesel additive. Once released into the atmosphere, 2-PE undergoes degradation processes mainly by reactions with oxidants (OH and NO_3_ radicals, Cl atoms). This study provides a kinetic and mechanistic investigation of the atmospheric degradation of 2-PE using FTIR (Fourier transform infrared spectroscopy) and GC-MSTOF (gas chromatography/mass spectrometry time of flight). Rate coefficients at ambient temperature and pressure, were (units cm^3^ molecule^–1^ s^–1^): (1.99 ± 0.12) × 10^–10^, (2.54 ± 0.15) × 10^–11^ and (7.20 ± 0.53) × 10^–15^ for Cl, OH^•^ and NO_3_^•^ reactions, respectively. Detected products include formaldehyde, acetaldehyde, propyl formate, 2-hydroxyethyl formate and 2-hydroxyethyl propanoate, quantified by FTIR, and nitrated compounds detected in the presence of NO_*x*_. Based on product distributions and structure–reactivity relationships, two predominant reaction pathways are proposed, both initiated by the oxidant attack on the –CH_2_– group adjacent to the ether. Atmospheric lifetimes indicate that OH^•^ reaction dominates the 2-PE removal. Estimated GWP and ozone formation indices confirm that 2-propoxyethanol is a short-lived VOC with limited direct climate impact, while providing a basis for comparison with structurally related glycol ethers and other VOCs.

## Graphical abstract

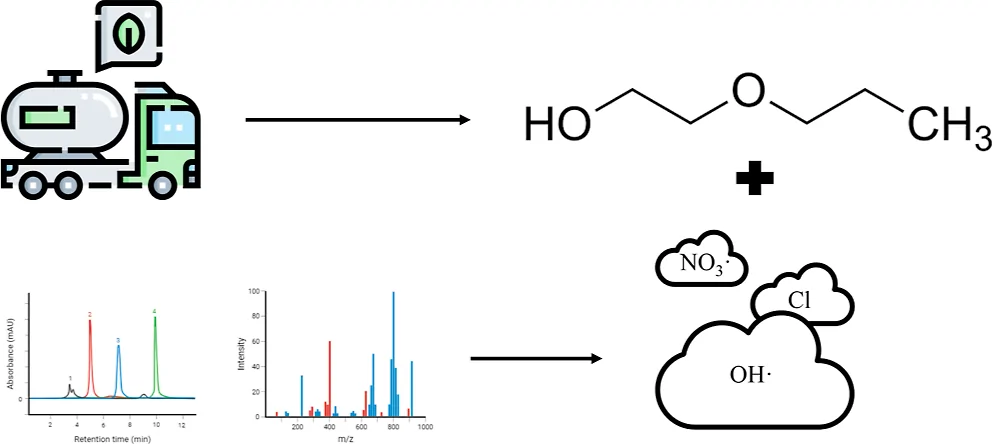

## Introduction

Hydroxy ethers (HEs), also referred to as glycol ethers when derived from ethylene or propylene glycol, are oxygenated volatile organic compounds (OVOCs) containing both hydroxyl and ether functionalities. Their extensive industrial relevance as solvents and chemical intermediates spans coatings, pharmaceuticals, cosmetics, and increasingly the automotive and aerospace sectors, where they are incorporated into antifreeze, cleaners, and specialty fluids.^1^ Some HEs have even been considered as components in diesel fuel formulations to improve combustion properties^2^

In recent years, hydroxy ethers have gained attention as emerging atmospheric contaminants, due to their increasing production, widespread use, and the limited understanding of their environmental and health impacts. Among these compounds, and according to the Haz-Map database (NIOSH), which compiles occupational hazard information from governmental and scientific sources, 2-propoxyethanol is associated with occupational exposure in industrial and consumer product use.^3^ Although quantitative atmospheric emission inventories for individual glycol ethers such as 2-propoxyethanol are not available, these compounds are widely used in industrial and consumer applications associated with indoor and outdoor VOC emissions. For this class of compounds, emissions linked to consumer product use have been estimated at approximately 32 tonnes day^–1^ in California, corresponding to about 1 g person^–1^ day^–1^ of emitted organic compounds and inhalation intakes on the order of 10 mg person^–1^ day^–1^.^4^ Although these values are not compound-specific, they provide an indication of the potential atmospheric relevance of hydroxyethers. Market reports aimed at industrial and commercial sectors indicate large-scale global production and consumption of glycol ethers (e.g., EMR, 2025) underscoring the widespread industrial relevance of this solvent class and the importance of understanding their atmospheric fate.

Once emitted into the atmosphere, 2-PE, like other organic compounds, may degrade via reactions with oxidants such as OH and NO_3_ radicals and Cl atoms, being ozone degradation negligible. While several studies have quantified its reactivity with OH radicals,^5,6^ no kinetic or mechanistic data are available for its reactions with Cl or NO_3_^•^. Direct photolysis of 2-PE was neglected, as saturated alcohol and ether functionalities do not exhibit significant absorption in the actinic UV region. This assumption is based on analogy with structurally similar oxygenated VOCs and is consistent with atmospheric chemistry reviews identifying radical-initiated reactions as the dominant loss processes^7^

Because atmospheric transformations of OVOCs can generate secondary pollutants with adverse environmental and health effects, such as carbonyls, organic acids, and secondary organic aerosols, understanding the behavior of 2-PE is important within the broader context of air quality and human exposure. This study provides a kinetic and mechanistic investigation of the atmospheric degradation of 2-PE using Fourier transform infrared spectroscopy (FTIR) and gas chromatography/mass spectrometry time-of-flight (GC–MS-TOF). To evaluate its environmental relevance as an emerging contaminant of concern for air quality and health, we assess key parameters including atmospheric lifetime, deposition rates, Global Warming Potential (GWP), Photochemical Ozone Creation Potential (POCP), and the identity and significance of degradation products.

## Experimental Section

The experimental setup has been described in detail elsewhere.^8–10^ Briefly, the kinetics and products experiments were conducted in a cylindrical Pyrex reactor with a total volume of 50 L, connected to a Fourier Transform Infrared (FTIR) spectrometer (Thermo Nicolet 6700). Specifically, a Saturn Series Long Path gas cell (Model 50L/200 V) was employed. The cell is equipped with a multiple-reflection optical system consisting of three internal mirrors, allowing a variable optical path length of up to 200 m, which can be adjusted by micrometric displacement of the mirrors. The reactor is connected to a vacuum line to allow evacuation and introduction of reactants and is evacuated using a rotary pump (Telstar RD-18) protected by a liquid nitrogen trap. Pressure inside the chamber is continuously monitored using a capacitance manometer (Varian CeramiCel CDG gauge, 0–1000 Torr range). The cell is also equipped with a septum port that enables sampling using solid-phase microextraction (SPME) fibers when product analysis is required.

Infrared radiation is directed into and out of the reactor through an external optical box coupled to the FTIR spectrometer. The spectrometer is equipped with a liquid-nitrogen-cooled MCT/A detector (mercury cadmium telluride, HgCdTe). The windows of the cell that allow the transmission of infrared radiation are made of zinc selenide (ZnSe). The entire reactor is enclosed in an aluminum housing to prevent exposure to ambient laboratory light. For experiments requiring photolysis, the chamber is surrounded by eight actinic lamps (Philips TL-K 4W, Actinic BL), emitting in the 350–400 nm range (λ_max_ ≈ 360 nm).

Complementary product analysis was also performed using gas chromatography combined with a time of flight mass spectrometer (GC–MSTOF). Sampling was performed using solid phase microextraction (SPME) with a DVB/CAR/PDMS fiber, followed by thermal desorption in the GC injection port. An Equity-1701 capillary column was employed for chromatographic separation. The operating conditions were as follows: injector temperature set at 250 °C, interface at 200 °C, and an oven temperature program beginning at 40 °C, increasing to 120 °C, and subsequently to 200 °C.

Rate coefficients were obtained using the relative rate method, that consist in determining the rate coefficient of the reaction (*k*_2‑PE_) between 2-PE and a given oxidant (X) by comparison with a reference reaction of the same oxidant with a compound of known rate coefficient (*k*_R_).

1 $${X+2}_{-}PE\to products\left(k_{2-PE}\right)$$

2 $$X+R\to products\left(k_{R}\right)$$

The method assumes that the only significant loss processes for both 2-PE and *R* are their reactions with the oxidant X. The integration of the corresponding rate equations leads to the following eq 3

3 $$\ln\left(\frac{{\left[2-PE\right]}_{o}}{{\left[2-PE\right]}_{t}}\right)=\frac{k_{2-PE}}{k_{R}}\ln\left(\frac{{\left[R\right]}_{o}}{{\left[R\right]}_{t}}\right)$$

where [2-PE]_0_ and [*R*]_0_ are the initial concentrations of the hydroxyether and reference, and [2-PE]_*t*_ and [*R*]_*t*_ are the corresponding concentrations at reaction time *t*. Plots of ln­([2-PE]_0_/[2-PE]_*t*_) versus ln­([*R*]_0_/[*R*]_*t*_) yielded linear fits with zero intercept, and slopes provided the rate coefficient of 2-PE (*k*_2‑PE_), using the known *k* of the reference compound (*k*_*R*_). Experiments involved reactions with OH and NO_3_ radicals and Cl atoms, generated in situ as previously described:^9,10^ OH^•^ from CH_3_ONO photolysis (λ = 354 nm), Cl atoms from Cl_2_ photolysis (λ = 360 nm), and NO_3_^•^ from N_2_O_5_ thermal decomposition. In the OH generation system based on methyl nitrite photolysis, CH_3_ONO photolysis initially produces CH_3_O and NO radicals, which in the presence of O_2_, are rapidly converted through well-established reaction pathways leading to efficient OH formation and limiting the reactivity of secondary radical species;^11^ therefore, air was used as carrier gas. In contrast, NO_3_ radicals and Cl atoms were generated in high-purity N_2_ to avoid oxygen-driven secondary reactions which could introduce additional propagation pathways and complicate kinetic interpretation. The choice of carrier gas (N_2_ or air) does not affect the measured bimolecular rate coefficients, since N_2_ and O_2_ exhibit comparable third-body efficiencies under atmospheric-pressure conditions.

For product analysis, the same procedure was applied using air and without reference compounds, since product studies aim to reproduce atmospherically relevant oxidation conditions; using air ensures the presence of O_2_ needed for the formation and stabilization of oxygenated products.

After introducing 2-PE, oxidant precursors, and reference compounds, the time-resolved IR spectra were recorded and processed using OMNIC software. 2-PE was monitored using its C–O stretching absorption bands at 1141 and 1071 cm^–1^, as well as the O–H stretching band at 3638 cm^–1^. Reference compounds were monitored using characteristic infrared absorption bands: aldehydes (hexanal and propanal) at the C–H stretching region of the –CHO group (∼2700 cm^–1^), cyclohexane at the C–H stretching band (2934 cm^–1^), and 1-butene at the out-of-plane =C–H bending vibration (912 cm^–1^) and the =C–H stretching vibration (3090 cm^–1^).

Typical concentrations were: (a) For kinetic experiments: 8–13 ppm for 2-PE and 5–13 ppm for references, 12–40 ppm of Cl_2_, 9–43 ppm of CH_3_ONO, 9–50 ppm of NO, and 6–52 ppm of N_2_O_5_. Conversion levels ranged from 9–46% (Cl), 6–24% (OH^•^), and 10–30% (NO_3_^•^). Experiments were performed at 706 ± 11 Torr and 298 ± 2 K; b) For products experiments: 9–28 ppm for 2-PE, 18–23 ppm of Cl_2_, 21–23 ppm of CH_3_ONO, 21–26 ppm of NO (Cl experiments), 19–22 ppm of NO (OH^•^ experiments) and 14–20 ppm of N_2_O_5_.

Reagents included 2-PE (99%) and reference compounds (cyclohexane (99%), hexanal (98%), propanal (97%) and 1-butene (≥97%)) from Sigma-Aldrich. For product identification: Propyl formate (97%) from Sigma-Aldrich, 2-hydroxyethyl formate (95%) from Indagoo, and 2-hydroxyethyl propanoate (95%) from Biosynth. CH_3_ONO and N_2_O_5_ were synthesized following standard procedures.^11,12^ Other gases: Cl_2_ (Praxair, 99%) NO (Air liquid, 98.5%), N_2_O_4_ (Fluka, 99.5%), N_2_, and synthetic air (Praxair, 99,999%).^13^

## Results and Discussion

### Kinetic Study

Prior to performing the kinetic studies, a series of preliminary experiments was carried out to evaluate the possible occurrence of secondary processes that could interfere with the main reaction, such as heterogeneous losses on the reactor walls of either 2-PE or reference compounds, photolysis induced by the radiation emitted from the lamps, and potential reactions between the compounds and the radical precursor. When present, such secondary processes would require appropriate corrections in the determination of the relative rate coefficients. However, under the experimental conditions employed in this work, these effects were assessed and found to be negligible. Additionally, it was verified that no overlap was observed between the infrared absorption bands used for monitoring the compounds of interest.

Figure 1 shows kinetic plots for the Cl + 2-PE reaction; analogous plots for OH^•^ and NO_3_^•^ reactions are provided in the Supporting Information (Figures S1 and S2). The slopes of the linear fits, combined with the literature rate coefficients of the reference compounds (eq 3), were used to derive the rate coefficients for 2-PE. Near-zero intercepts were obtained in all cases, indicating negligible influence of secondary chemistry. When multiple reported values were considered, weighted average rate coefficients were used to provide representative values. Each individual measurement was weighted according to its associated uncertainty, so that studies with lower reported uncertainties contributed more strongly to the resulting average. This approach reduces the influence of less precise determinations and yields mean values that better reflect the most reliable available kinetic data. Final rate coefficients calculated as weighted averages are summarized in the Table 1. An explanation of these calculations is included in the Supporting Information.

**Figure 1:**
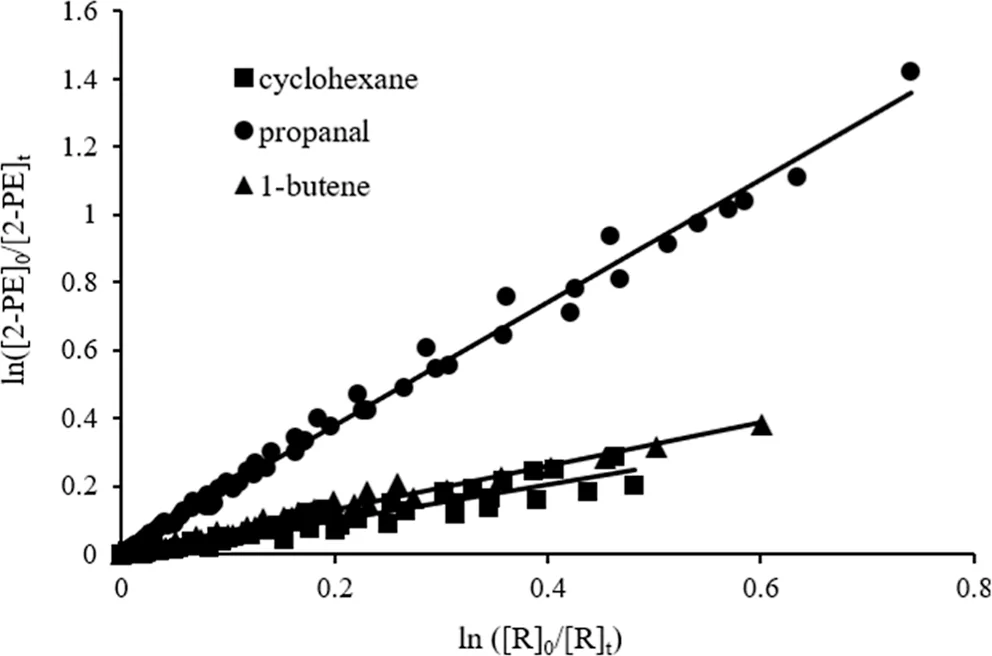
Plot according to eq 3 for the reaction of 2-PE with Cl atoms with three reference compounds.

**Table 1:** Summary of Relative Rate Coefficients and Absolute Rate Coefficients for Reactions of 2-PE with Cl Atom1s and OH and NO_3_ Radicals^e^

reaction	reference compound^d^	Run	*k*_2‑PE_/*k*_ref_ ± 2σ	*k*_2‑PE_ ± 2σ^a^ ^,^ ^b^ ^,^ ^c^
2-PE + Cl^a^	1-butene	1	0.80 ± 0.04	2.40 ± 0.49
	*k*_Cl_ = 3.00 ± 0.60	2	0.64 ± 0.01	1.92 ± 0.39
		3	0.71 ± 0.03	2.13 ± 0.43
	propanal	1	1.75 ± 0.06	2.19 ± 0.44
	*k* = 1.25 ± 0.25	2	1.87 ± 0.04	2.34 ± 0.47
		3	2.05 ± 0.03	2.56 ± 0.52
		4	2.06 ± 0.09	2.58 ± 0.53
	cyclohexane	1	0.44 ± 0.09	1.45 ± 0.38
	*k* = 3.30 ± 0.50	2	0.49 ± 0.02	1.62 ± 0.25
		3	0.62 ± 0.03	2.05 ± 0.32
	weighted average			1.99 ± 0.12
2-PE + OH^b^	1-butene *k* = 3.10 ± 0.62	1	0.72 ± 0.04	2.23 ± 0.47
		2	0.93 ± 0.07	2.88 ± 0.62
		3	1.01 ± 0.03	3.13 ± 0.63
	hexanal *k* = 2.85 ± 0.43	1	0.81 ± 0.05	2.31 ± 0.48
		2	0.92 ± 0.06	2.62 ± 0.44
		3	0.93 ± 0.05	2.65 ± 0.32
	propanal *k* = 1.99 ± 0.30	1	1.57 ± 0.06	3.12 ± 0.39
		2	1.42 ± 0.04	2.83 ± 0.41
		3	1.03 ± 0.08	2.05 ± 0.46
	weighted average			2.54 ± 0.15
2-PE + NO_3_ ^c^	1-butene *k* = 13.00 ± 3.25	1	0.62 ± 0.03	8.06 ± 2.03
		2	0.65 ± 0.02	8.45 ± 2.15
		3	0.51 ± 0.02	6.63 ± 1.69
	propanal *k* = 6.30 ± 2.52	1	0.97 ± 0.05	6.11 ± 2.41
		2	0.98 ± 0.06	6.17 ± 2.51
		3	0.88 ± 0.06	5.54 ± 2.24
	hexanal	1	0.49 ± 0.03	7.89 ± 1.26
	*k* = 16.1 ± 2.40	2	0.45 ± 0.02	7.25 ± 1.12
		3	0.44 ± 0.03	7.08 ± 1.16
	weighted average			7.20 ± 0.53

a Rate coefficients (*k*) are given in cm^3^ molecule^–1^ s^–1^ and reported as × 10^–10^, including the corresponding reference rate coefficients.

b Rate coefficients (*k*) are given in cm^3^ molecule^–1^ s^–1^ and reported as × 10^–11^, including the corresponding reference rate coefficients.

c Rate coefficients (*k*) are given in cm^3^ molecule^–1^ s^–1^ and reported as × 10^–15^, including the corresponding reference rate coefficients.

d Values from.^13^

e The rate coefficients used for the reference compounds are also listed in the table.

The overall uncertainty σ­(*k*_2‑PE_) results from the combined contributions of the slope error (σ_slope_) and the reference rate uncertainty (σ_*kR*_), as given in eq 4

4 $$\sigma \left(k_{2-PE}\right)=\sqrt{{\left(k_{R}\times \sigma_{slope}\right)}^{2}+{\left(slope\times \sigma_{k_{R}}\right)}^{2}}$$

### Reactions with Cl

The reaction of 2-PE with Cl atoms was found to be very fast, with a rate coefficient of (1.99 ± 0.12) × 10^–10^ cm^3^ molecule^–1^ s^–1^. As expected for chlorine atom reactions with oxygenated volatile organic compounds (OVOCs), the measured rate coefficient is close to the gas-kinetic limit and shows only weak dependence on molecular structure.^10,14–17^

Available kinetic data for Cl-initiated oxidation of hydroxyethers are limited.^8–10,18,19^ The value obtained here is in good agreement with reported rate coefficients for structurally related compounds such as 2-ethoxyethanol (1.95 ± 0.10 × 10^–10^ cm^3^ molecule^–1^ s^–1^;^10^) and 2-isopropoxyethanol (2.18 ± 0.15 × 10^–10^ cm^3^ molecule^–1^ s^–1^;^8^). Minor variations can be attributed to differences in alkyl chain length rather than specific functional group effects.

These results confirm that 2-PE will undergo rapid oxidation in environments influenced by reactive chlorine, such as coastal or polluted regions, implying a short atmospheric persistence under such conditions.

### Reaction with OH Radicals

The rate coefficient for the OH^•^ + 2-PE reaction was determined to be (2.54 ± 0.15) × 10^–11^ cm^3^ molecule^–1^ s^–1^. This value is slightly higher than that reported by,^5^ (2.14 ± 0.6) × 10^–11^ cm^3^ molecule^–1^ s^–1^ but agrees within the combined uncertainties. It is approximately 35% higher than the value reported by,^6^ (1.64 ± 0.07) × 10^–11^ cm^3^ molecule^–1^ s^–1^, who employed a single reference compound and noted possible systematic uncertainties.

For hydroxyethers, OH-initiated oxidation proceeds predominantly via H-abstraction from primary or secondary carbon atoms adjacent to the ether oxygen. Reported OH^•^ rate coefficients generally increase with increasing alkyl chain length attached to the ether group.^5,6,8–10,18–29^ The rate coefficient obtained here follows this trend, lying between those reported for 2-ethoxyethanol (averaged value of 1.66 × 10^–11^ cm^3^ molecule^–1^ s^–1^) and 2-butoxyethanol (2.94 × 10^–11^ cm^3^ molecule^–1^ s^–1^,^22^).

For OH-initiated oxidation of oxygenated volatile organic compounds, including alcohols, ethers and hydroxyethers, it is well established that weakly bound hydrogen-bonded or van der Waals prereactive complexes play a key mechanistic role in controlling the reaction kinetics.^30,31^ Previous studies have shown that these complexes form prior to hydrogen atom abstraction, influencing molecular orientation, reaction site selectivity and the effective activation barrier. In alcohols, the presence of the hydroxyl group not only enhances the formation of such prereactive complexes but also electronically activates adjacent C–H bonds through inductive effects, resulting in weakened C–H bond strengths and stabilization of the abstraction transition state, which leads to pronounced site-specific reactivity and, in many cases, non-Arrhenius temperature dependences^32,33^ Similar considerations can apply to ethers and hydroxyethers, where one or more oxygen atoms increase molecular polarity and favor the formation of prereactive complexes with OH radicals. In these cases, OH reactivity can be described by electronic effects associated with the oxygen atom and by molecular conformation, which together govern the accessibility of abstractable hydrogen atoms within prereactive complexes and the favorability of reactive orientations.^34,35^

### Reaction with NO_3_ Radicals

The reaction of 2-PE with NO_3_^•^ radicals yielded a rate coefficient of (7.20 ± 0.53) × 10^–15^ cm^3^ molecule^–1^ s^–1^. To our knowledge, no previous kinetic data are available for the NO_3_-initiated oxidation of 2-PE, and the present study therefore provides the first determination of its nighttime reactivity.

Available data for NO_3_ reactions with hydroxyethers are scarce but reported rate coefficients generally increase with alkyl chain length adjacent to the ether group.^9,10,22,36,37^ The value obtained here is higher than that reported for 2-ethoxyethanol (5.86 ± 0.33 × 10^–15^ cm^3^ molecule^–1^ s^–1^;^10^), but also exceeds those reported for 2-butoxyethanol (∼3 × 10^–15^ cm^3^ molecule^–1^ s^–1^) (averaged from^22,36,37^ ) and a more substituted compounds such as 2-isopropoxyethanol (3.16 ± 0.45 × 10^–15^ cm^3^ molecule^–1^ s^–1^;^8^). This behavior, not observed for OH^•^ or Cl reactions, may reflect that NO_3_ reactions are less influenced by hydrogen-bonded prereactive complexes, owing to the larger size and different electronic structure of the radical. Therefore, geometric accessibility and conformational effects play a more important role in determining which abstraction sites are preferentially accessed during reactive collisions. Under these conditions, steric hindrance should be considered not as an independent controlling factor, but rather as a limiting factor that restricts access to electronically activated hydrogen atoms, particularly as substitution around the oxygen atom increases. This could explain why reactivity trends for NO_3_ with hydroxyethers do not follow a simple monotonic increase with alkyl chain length. Instead, a maximum reactivity can be observed for moderately substituted compounds, such as 2-propoxyethanol, while further substitution leads to decreased reactivity due to increasing steric hindrance.

### Kinetic Comparison among Atmospheric Oxidants

The reactivity trend observed: *k*_Cl_ > *k*_OH_ > *k*_NO3_, is consistent with the molecular geometry and electron density of each oxidant, as well as with trends previously reported for structurally related hydroxyethers (e.g.,^10^). Rate coefficients also highlights that 2-PE reacts efficiently with the main atmospheric oxidants, information that is relevant for determining its atmospheric lifetime and assessing its behavior as an oxygenated volatile organic compound (OVOC) of emerging environmental interest.

### Influence of Functional-Groups on Molecular Reactivity

More information about 2-PE reactivity can be obtained by analyzing the influence of functional-groups on molecular reactivity. Table 2 provides a comparative overview of reported rate coefficients for reactions of OH^•^, Cl and NO_3_^•^ with C_5_ compounds with different terminal functional groups, selected to maintain a comparable number of CH_2_ abstraction sites. Relative to alkanes, introducing either –OH or –O– functional groups increases reactivity toward OH^•^ by half an order of magnitude, as observed for 1-pentanol and ethyl propyl ether. Combining both groups (as in 2-PE) yields the largest enhancement. For NO_3_^•^, the activating effect is even stronger, approaching 2 orders of magnitude, indicating that NO_3_ reactivity is particularly sensitive to the presence and nature of functional groups. This reflects the significant activating role of hydroxyl and ether groups compared to the corresponding alkane. Rate coefficients for Cl atom reactions show comparatively smaller variations, consistent with the lower selectivity of this radical. This behavior is characteristic of OVOCs and suggests a potential role of 2-PE in the formation of oxygenated secondary pollutants.

**Table 2:** Comparison of Reported Bimolecular Rate Coefficients for Reactions of OH, NO_3_ and Cl with C_5_ Compounds with Different Terminal Functional Groups

compound	structure	*k*_Cl_ ± 2σ/10^–10^	*k*_OH_ ± 2σ/10^–11^	*k*_NO3_ ± 2σ/10^–15^
2-PE	CH_3_CH_2_CH_2_OCH_2_CH_2_OH	1.99 ± 0.12	2.54 ± 0.15	7.20 ± 0.53
pentane^a^	CH_3_CH_2_CH_2_CH_2_CH_3_	2.70 ± 0.41	0.38 ± 0.04	0.083 ± 0.02
1-pentanol^a^	CH_3_CH_2_CH_2_CH_2_CH_2_OH	2.40 ± 0.41	1.10 ± 0.17	-
ethyl propyl ether	CH_3_CH_2_CH_2_OCH_2_CH_3_	-	1.12 ± 0.17	-
pentanal	CH_3_CH_2_CH_2_CH_2_CHO	2.40 ± 0.36	2.66 ± 0.40	16.20 ± 3.24
1-pentene	CH_3_CH_2_CH_2_CH=CH_2_	4.20 ± 0.2	3.22 ± 0.02	15 ± 3.3
1,2-epoxyhexane	CH_3_CH_2_CH_2_CH_2_CH(O)CH_2_	1.64 ± 0.25	57.60 ± 11.52	-

a Recommended values from.^13^ Rate coefficients (*k*) are given in cm^3^ molecule^–1^ s^–1^.

With respect to carbonyl compounds and alkene, although they exhibit distinct dominant reaction pathways, their inclusion in the Table 2 provides a useful reference for comparing the magnitude of functional-group activation effects. As expected, pentanal exhibit strongly enhanced reactivity toward OH^•^ and NO_3_^•^, reflecting efficient hydrogen abstraction at the α-position adjacent to the carbonyl group. Similarly, terminal alkenes such as 1-pentene show elevated rate coefficients due to the availability of fast addition pathways, particularly for OH^•^ and NO_3_^•^. In the case of 1,2-epoxyhexane, which contains one additional carbon atom relative to the C_5_ compounds considered, very high OH rate coefficients reflect the combined effects of ring strain and distinct radical interaction pathways, which differ fundamentally from those governing hydrogen abstraction in acyclic oxygenated compounds.

#### Structure–Activity Relationship Calculations

To complement the experimental data, Structure–Activity Relationship (SAR) calculations^38^ were applied using literature-based reactivity factors. This approach uses kinetic databases to assign reactivity factors (F­(R)) to functional groups based on their position in the molecule. Detailed calculations and the values used for *k*_prim_, *k*_sec_, *k*_terc_ and k_OH_, and reactivity factors (F­(R)), are provided in the Supporting Information. The overall rate coefficient for a saturated organic compound is calculated using eq 5

5 $$k_{abs}=\sum k_{prim}F\left(X\right)+\sum k_{\sec}F\left(X\right)F\left(Y\right)+\sum k_{tert}F\left(X\right)F\left(Y\right)F\left(Z\right)+\sum k_{OH}$$

in this study, reactivity factors proposed by Kerdouci et al. (2011) were applied for Cl and OH^•^ reactions, while those from^39^ were used for NO_3_^•^ reactions. Predicted *k* values (cm^3^ molecule^–1^ s^–1^) are 2.56 × 10^–10^ (Cl), 2.49 × 10^–11^ (OH^•^), and 3.51 × 10^–15^ (NO_3_^•^). In addition, for the reaction with OH radicals, an alternative SAR approach developed by Jenkin et al.^40,41^ This method is also based on site-specific H-atom abstraction but incorporates parametrizations derived from a broader and more recent kinetic data set, and it is widely used in atmospheric chemical modeling. The rate coefficient predicted using the Jenkin SAR approach is *k*_OH_ = 2.14 × 10^–11^ cm^3^ molecule^–1^ s^–1^, in very good agreement with both the experimental value and the estimation based in Kwok and Atkinson, 1995.^38^ The detailed calculations are included in the Supporting Information.

Therefore, the agreement is excellent for Cl and OH^•^, while NO_3_^•^ rate coefficient is underestimated, consistent with previous findings for related compounds,^8–10^ indicating the need to refine NO_3_^•^ reactivity factors. In this context, Carter (2021)^42^ has recently proposed an updated SAR formulation for abstraction reactions, reformulating the calculation of global abstraction rate coefficients and revising several key parameters. Using this, the predicted rate coefficients (cm^3^ molecule^–1^ s^–1^) for the reactions of 2-propoxyethanol are 2.18 × 10^–10^ for Cl atoms, 2.9 × 10^–11^ for OH radicals, and 6.02 × 10^–15^ for NO_3_ radicals. The most significant improvement with respect to earlier SAR estimations is observed for the NO_3_ radical reactions, for which the updated approach yields values substantially closer to the experimental rate coefficient, confirming that the revised parametrization proposed by Carter (2021)^42^ provides a markedly improved description of NO_3_ radical reactivity for oxygenated compounds such as glycol ethers.

### Products Study and Mechanism

#### Overview

To analyze the reaction products, an initial mechanism was proposed based on established atmospheric oxidation principles^43^ and literature on analogous hydroxyethers. Because these compounds lack double bonds, oxidation by all radicals begins with hydrogen abstraction. SAR calculations (see Supporting Information) indicate negligible attack at the –CH_3_ and –OH positions (1 and 6 positions). Accordingly, the four feasible pathways and the predicted attack fractions for each oxidant (calculations provided in the Supporting Information) are the corresponding to the H-abstraction in the carbons 2, 3, 4 and 5, leading to the four possible pathways included in the Scheme 1.

**Scheme 1:**
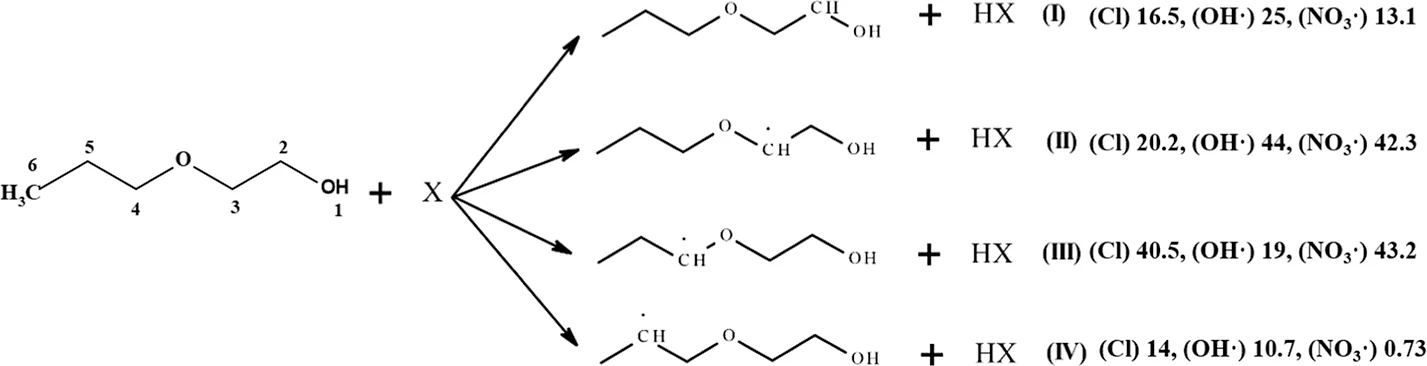
Reaction pathways (I, II, III and IV) for the H-Abstraction of the oxidant (X = Cl, OH^•^ or NO_3_^•^) to the 2-PE Molecule, and Attack Fractions (in %) Predicted by SARs

The percentages indicated in the scheme represent the relative contributions of H-atom abstraction at each carbon atom, as predicted by the SAR calculations, and are given separately for each oxidant (Cl, OH^•^ or NO_3_^•^). Full details are provided in the Supporting Information. The calculated attack fractions indicate that pathways II and III dominate the initial oxidation.

After initial H-abstraction, the resulting alkyl radical rapidly reacts with O_2_ to form a peroxy radical (RO_2_^•^). The subsequent RO_2_ chemistry follows the established atmospheric pathways governed by ambient NO levels, highlighting the role of 2-PE as an OVOC whose degradation products can vary substantially between NO-rich and NO-poor environments. In NO-free conditions, RO_2_^•^ undergoes either radical self-reaction (yielding RO^•^ + O_2_) or a molecular channel forming an alcohol and a carbonyl compound, while the ROOR-forming channel was not considered here. In the presence of NO, RO_2_^•^ primarily forms NO_2_ and RO^•^, with minor formation of alkyl nitrates (RONO_2_). These considerations support the mechanism outlined in Scheme 2, with experimental work aimed at identifying products and determining the dominant pathways.

**Scheme 2:**
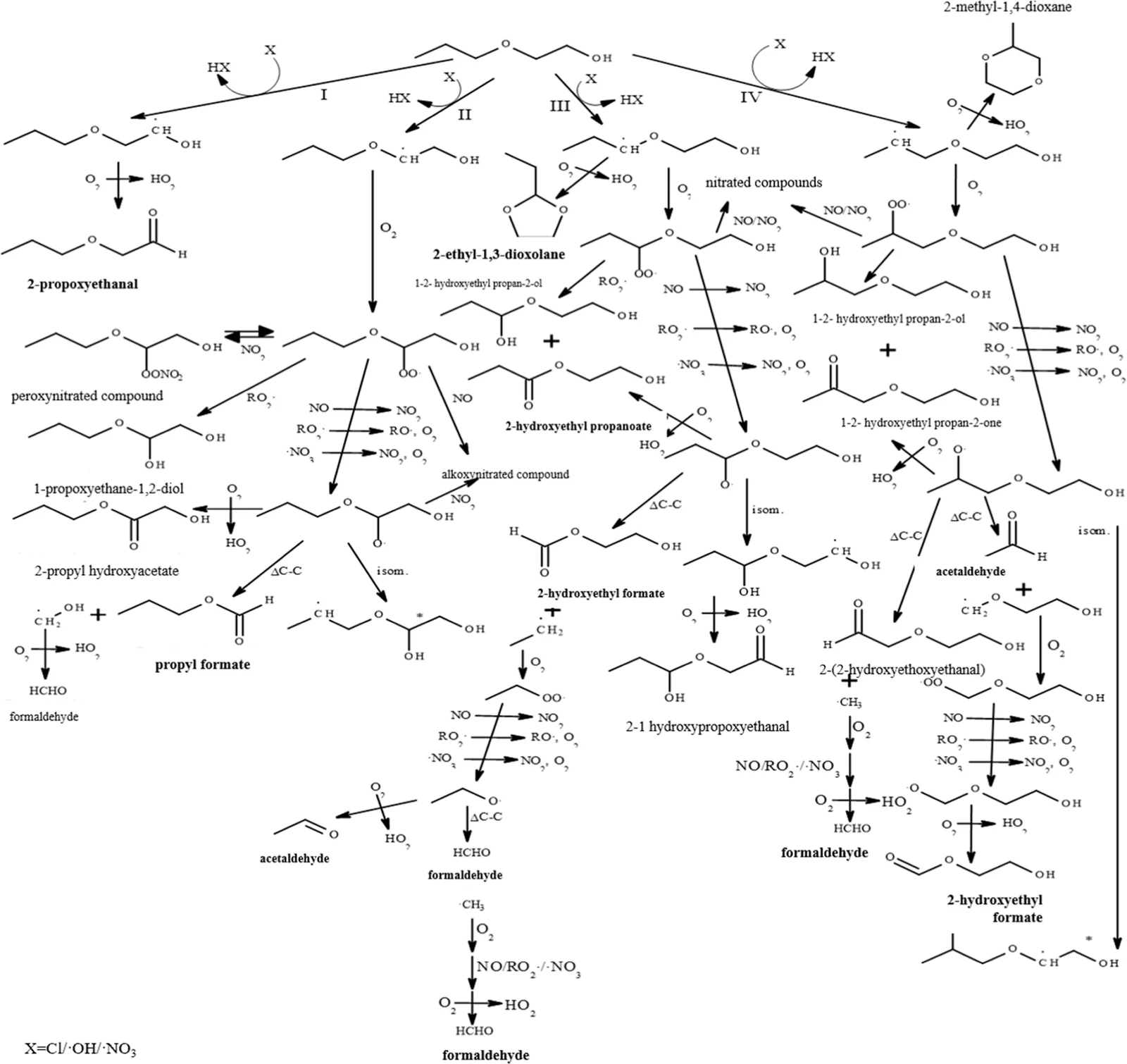
Proposed Mechanism of the Reactions of 2-PE with the Atmospheric oxidants Cl Atoms, OH and NO_3_ Radicals^a^

a The products identified are shown in bold.

Reaction products from the oxidation of 2-propoxyethanol (2-PE) were investigated using complementary FTIR and SPME/GC–MS techniques. FTIR spectroscopy was employed for time-resolved identification and quantification, while GC–MS provided additional qualitative identification. Although the same analytical approach was applied for all oxidants, the nature and relative importance of products varied depending on whether OH radicals, Cl atoms, or NO_3_ radicals initiated the oxidation.

Figure 2 displays the residual FTIR spectra for the reactions of 2-PE with OH, NO_3_ radicals, and Cl atoms after ∼20% consumption of reactant. Spectra were obtained by subtracting contributions from 2-PE, oxidant precursors, NO_2_, and HNO_3_. Reference spectra of expected products (propyl formate, acetaldehyde, formaldehyde) are included for comparison.

**Figure 2:**
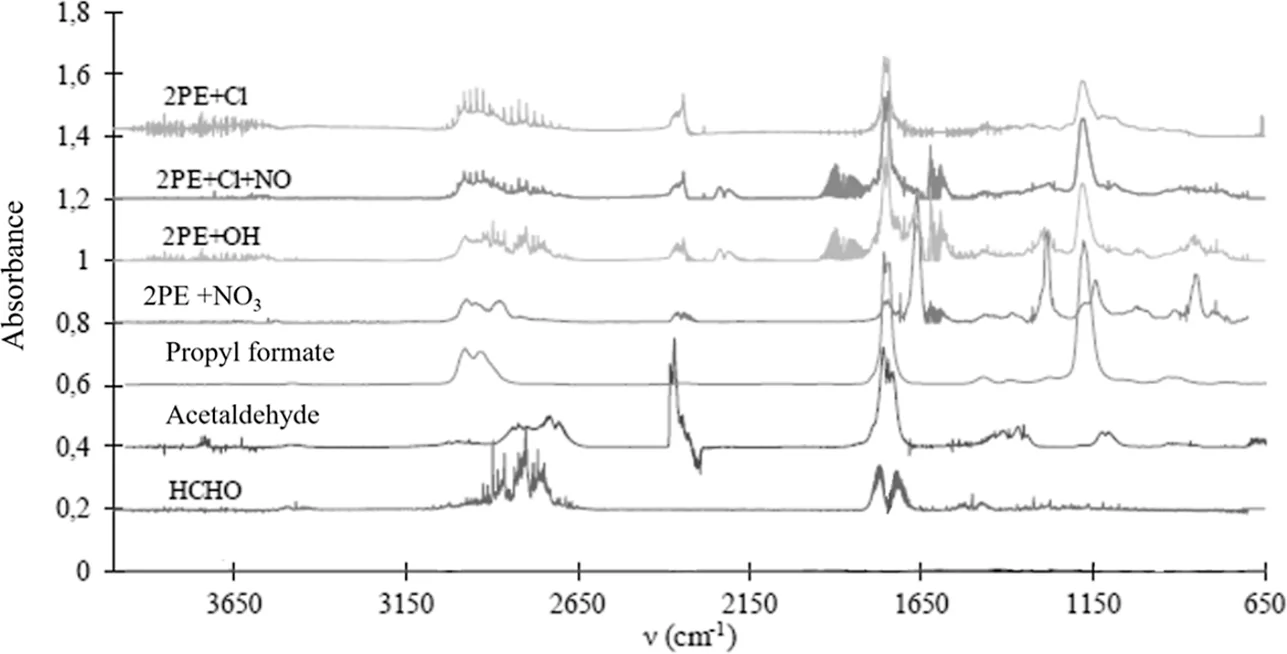
Residual FTIR spectra of the reactions of 2-PE (20% consumed) with the different atmospheric oxidants. Included in the figure the reference FTIR spectra of propyl formate, acetaldehyde and HCHO.

Common FTIR bands are observed in reactions with different oxidants, including carbonyl stretching bands (C=O, ∼1750 cm^–1^), C–O stretching vibrations (1100–1300 cm^–1^) and aliphatic C–H stretching bands (2700–3000 cm^–1^). In NO_3_^•^ reactions, characteristic alkyl-nitrate bands (RONO_2_ at ∼1683, 1283, 845 cm^–1^) and weaker peroxynitrates features (ROONO_2_ at ∼1718, 1300, and 793 cm^–1^) are observed, confirming that nitrate-radical chemistry contributes to nitrogen-containing secondary products. A distinct band at ∼1140 cm^–1^ and differences in the C–H region further distinguish this spectrum.

Formaldehyde is clearly identified in Cl and OH^•^ reactions,^44^ consistent with its coproduction with propyl formate via pathway II. Propyl formate bands are consistently observed for all oxidants, confirming its formation and indicating that H-abstraction adjacent to the ether oxygen is a relevant pathway for 2-PE oxidation under atmospheric conditions. Acetaldehyde bands are not clearly observed, most likely due to spectral interference and the limited sensitivity of FTIR spectroscopy for this compound under the experimental conditions.

In Cl + NO experiments, peroxynitrated IR bands appear once 2-PE consumption exceeds 45%. At these conversions, bands of O_3_ and N_2_O_5_ are also detected, indicating the onset of secondary reactions. NO_2_ bands increase for ∼16 min and subsequently decrease as nitrated-product bands intensify.

### Experiments with SPME/GC–MS

Figure 3 shows representative chromatograms for 2-PE reactions with Cl atoms (with and without NO), OH, and NO_3_ radicals. For NO_3_-initiated experiments, interpretation is affected by an elevated baseline. All chromatograms show the unreacted 2-PE peak at *t*_*R*_ = 7.27 min and a consistent product peak at *t*_*R*_ = 7.84 min, whose mass spectrum (85% similarity) supports its tentative assignment to 2-hydroxyethyl formate (2-HEF).

**Figure 3:**
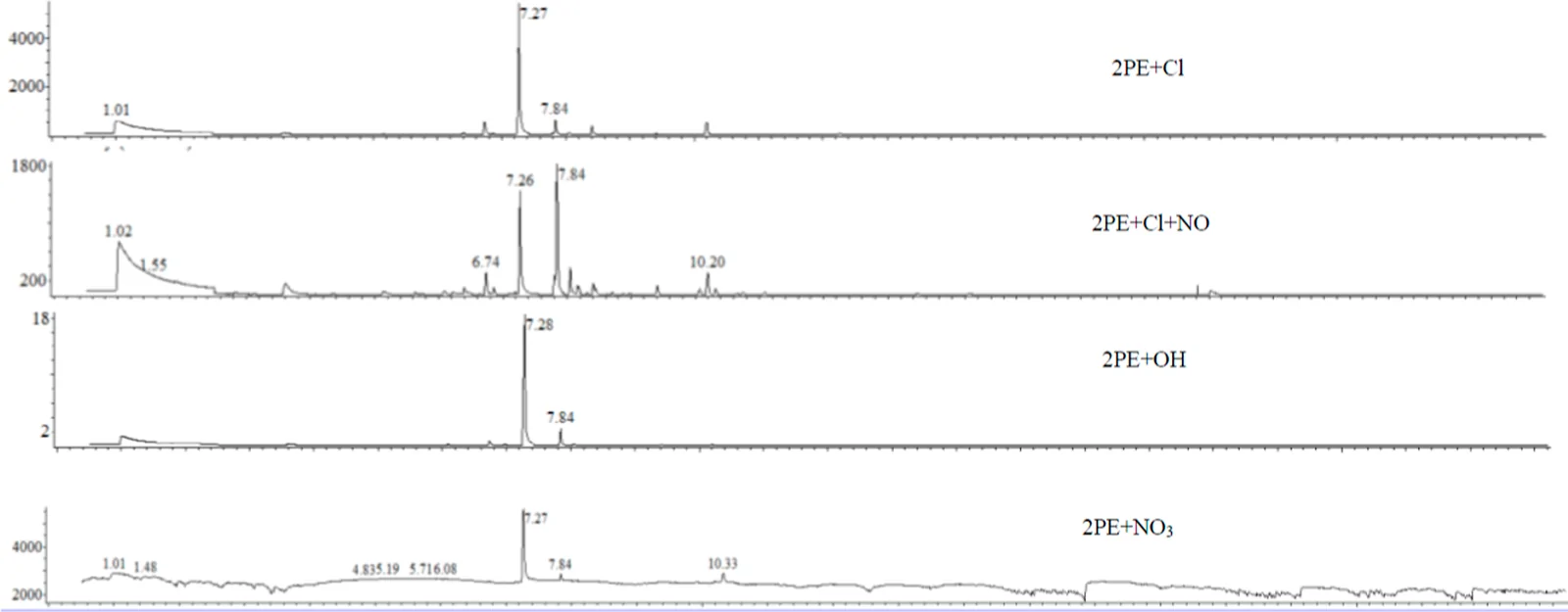
Chromatograms of the reaction of 2-PE with chlorine atoms in the absence of NO; in the presence of NO, with OH radicals and with NO_3_ radicals. The *Y* axis corresponds to the intensity of the peaks and the *X* axis corresponds to the chromatogram times indicated for each peak, the end of the chromatogram being 23.2 min.

In Cl-initiated reactions, additional peaks at *t*_*R*_ = 6.74 and 10.20 min indicate the formation of further products. The 6.74 min peak shows low spectral similarity (∼60%) to propanoic/isopropanoic acid, but it probably corresponds to an unidentified compound with a similar fragmentation pattern. The 10.20 min peak, observed in Cl and NO_3_^•^ reactions, matches 2-hydroxyethyl propanoate (95%) and is attributed to pathway III. A peak at *t*_*R*_ = 8.42 min, observed in Cl reactions without NO and at longer times with NO, may correspond to 2-hydroxyacetate propyl (similarity ∼30%) formed via pathway II.

In NO_3_ radical reactions, a peak at *t*_*R*_ = 10.33 min was detected and tentatively associated with isopropanoic acid, although its low spectral similarity (<50%) suggests that it correspond to an unidentified compound absent from the NIST database.^45^ A peak at *t*_*R*_ = 3.60 min, identified as propyl formate (95% similarity), and confirmed by FTIR, was observed from all oxidants (pathway II), although partially overlap with the air and are therefore not explicitly labeled in the representative chromatogram.

2-Propoxyethanal, previously reported by Markgraf et al. (1999),^5^ was not clearly identified, though the peak at *t*_*R*_ = 6.74 min could correspond to this compound (not commercially accessible and mass spectrum is not available in the equipment library). Low molecular weight aldehydes typically elute between 3 and 8 min. Examining the mass spectrum of this peak, it lacks the molecular ion (*m*/*z* ≈ 102) of 2-propoxyethanal, probably due to EI fragmentation, but shows fragments (*m*/*z* 87, 73, 59, 44, 29) consistent with the expected structure, supporting its tentative assignment (pathway I).

A peak at *t*_*R*_ = 6.09 min, observed in Cl + NO reactions and in OH^•^ reactions after more than 15 min, was assigned to 2-ethyl-1,3-dioxolane (66.7% similarity), a minor product previously proposed by Markgraf et al. (1999).^5^ Its formation involves O_2_ addition to the α-radical followed by intramolecular cyclization, though this pathway remains minor relative to the formation of 2-hydroxyethyl propanoate. Tables S2–S3 summarize peak assignments and EI mass spectral fragmentation patterns.

### Quantification of Products and Proposed Mechanism

Based on experimental results and SAR predictions (Table S4), OH-initiated oxidation occurs mainly via route II, forming propyl formate. Route III is also significant (19% for OH^•^ and more than 40% for Cl and NO_3_^•^ reactions, by SARs) and consistent with the observed formation of 2-hydroxyethyl formate and 2-hydroxyethyl propanoate. A smaller fraction (16, 25, and 13% for Cl, OH^•^, and NO_3_^•^ reactions respectively, by SARs) may proceed via route I, potentially yielding 2-propoxyethanal proposed by GC–MS.

To validate these findings, product quantification was performed by FTIR. Propyl formate, 2-hydroxyethyl formate, and 2-hydroxyethyl propanoate were quantified using commercial standards, while formaldehyde and acetaldehyde were quantified using Eurochamp reference spectra (,^46,47^ for formaldehyde;^48^ for acetaldehyde). Formaldehyde was not quantitatively determined in the OH-initiated experiments, as methyl nitrite (CH_3_ONO), used as the OH radical precursor, is a known source of HCHO upon photolysis.

For NO_3_-initiated reactions, nitrogen-containing products were quantified using an average integrated absorption coefficient of 1.2 × 10^–17^ cm molecule^–1^ in the 1250–1330 cm^–1^ region, as reported for similar compounds.^49^

Figure 4a displays FTIR time–concentration profiles for 2-PE and its products in OH-initiated reactions. Product yields were obtained by plotting concentration changes of quantified species against 2-PE loss (Figure 4b). Similar analyses for Cl, Cl + NO, and NO_3_^•^ reactions are presented in Figures S3–S5. For formaldehyde and acetaldehyde, yield reductions over time suggest secondary reactions of products with the oxidant. Accordingly, product yields were corrected following the formalism of Tuazon et al. (1986).^29^ Details of the correction procedure are provided in the Supporting Information.

**Figure 4:**
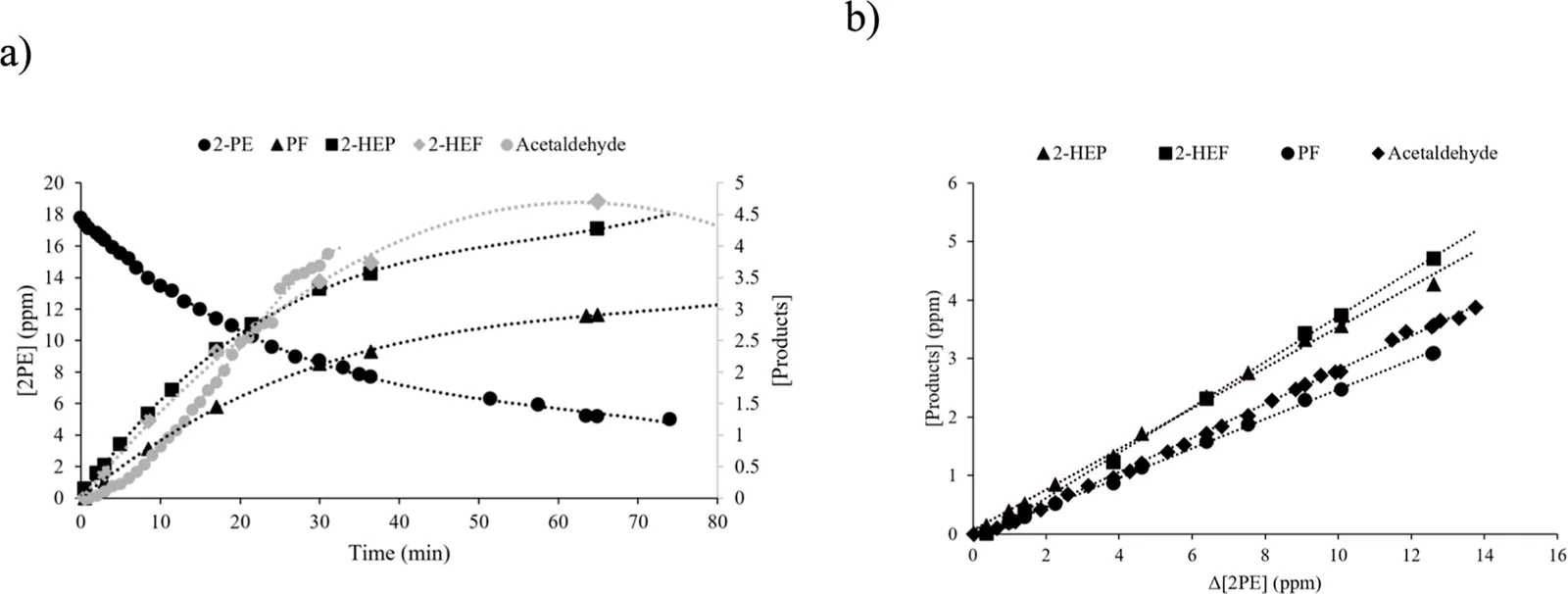
For OH^•^ reactions with 2-PE: (a) time–concentration profiles of reactant (2-PE) and products as measured by FTIR. (b) Plots of the variation of the concentration of the quantified products as a function of the 2-PE variation. [PF] = propyl formate; [2-HEP] = 2-hydroxy ethyl propanoate; [2-HEF] = 2-hydroxy ethyl formate.

Due to the complexity of the sample matrix (which contains a wide range of similar organic compounds), product yields were not determined using GC–MS and the analysis was therefore limited to qualitative identification. Figure 5 illustrates the time evolution of chromatographic peak areas for 2-PE and formed products in the Cl + 2-PE reaction without NO. Analogous profiles for other reactions are provided in Figures S6 and S7.

**Figure 5:**
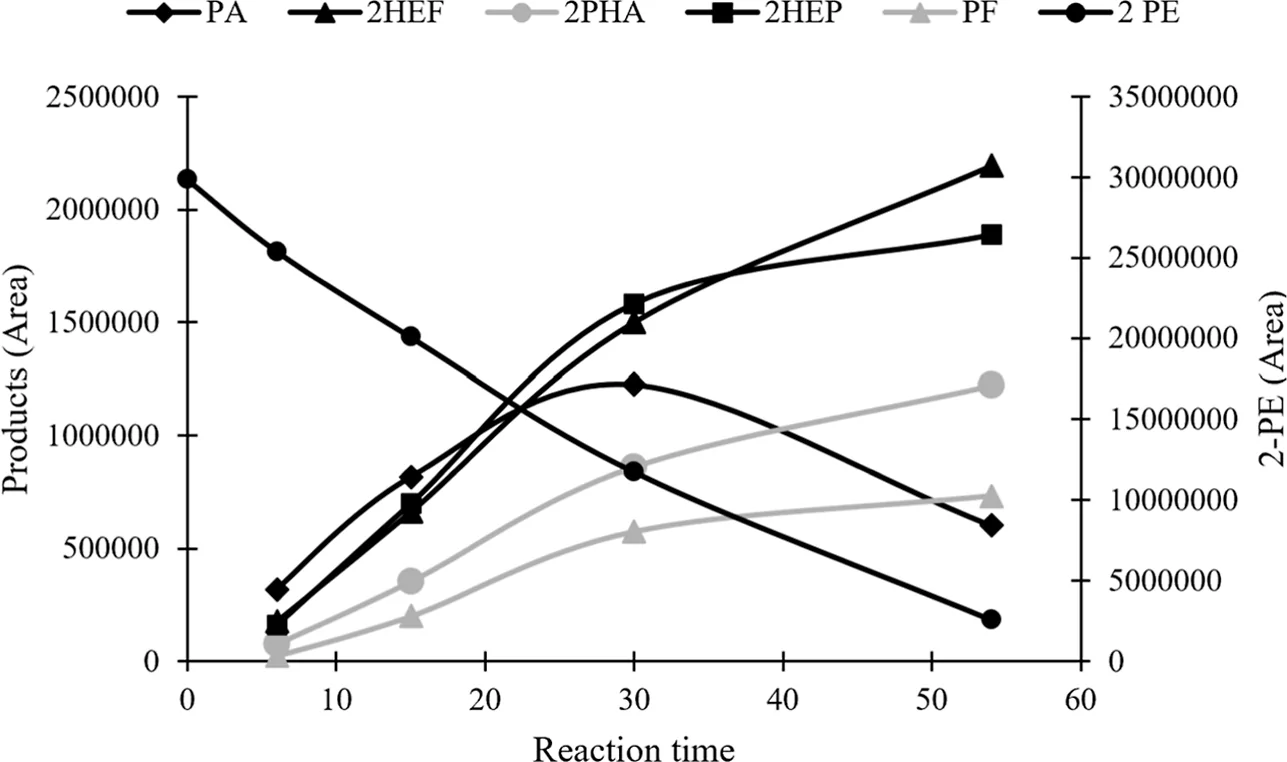
Plot of chromatographic areas of 2-PE and reaction products proposed (assigned to chromatographic peaks), as a function of the reaction time, for the 2-PE + Cl reaction. [PF] = propyl formate; [2HEP] = 2-hydroxy ethyl propanoate; [2HEF] = 2-hydroxy ethyl formate; [PA] = 2-propoxyethanal; [2PHA] = propyl hydroxyacetate.

Table 3 summarizes the average estimated product yields, with errors representing 2σ statistical uncertainties from regression of three replicates per reaction. Markgraf et al. (1999)^5^ investigated the OH^•^ + 2-PE reaction using GC–MS for identification and FID for quantification, reporting yields of 47% for propyl formate, 15% for 2-propoxyethanal, and 5% for 2-ethyl-1,3-dioxolane.

**Table 3:** A Summary of the Averaged Estimated Yields (in %) of Reaction Products Identified in This Work for the Reaction of 2-PE with OH^•^, Cl and NO_3_^•^: Formaldehyde (HCHO), Acetaldehyde (CH_3_CHO), Propyl Formate (PF), 2-Hydroxyethyl Formate (2HEF), 2-Hydroxyethyl Propanoate (2HEP) and Nitrated Compounds (Nitra. Comp)^a^

reaction	products					
	CH_3_CHO	HCHO	PF	2HEF	2HEP	nitra. comp
2-PE + Cl	9.9 ± 3.0	17.8 ± 2.8	12.5 ± 1.2	19.9 ± 1.6	18.7 ± 1.2	
TC(%)	48.2					
2-PE + Cl + NO	24 ± 1.3	22.9 ± 3.1	20.6 ± 0.9	36.2 ± 1.4	32.4 ± 0.7	
TC(%)	84.8					
2-PE + OH^•^	30 ± 3.2		26.1 ± 2.0	38.6 ± 1.2	36 ± 2.6	
TC(%)	92.1					
2-PE + NO_3_^•^		7.8 ± 8.4	6.5 ± 1.2	7.4 ± 4.3	4.4 ± 2.0	64 ± 42
TC(%)	16*					

a + Without nitrated.

In the present study, FTIR-based results yield a lower propyl formate fraction (26.1%) and reveals the formation of 2-hydroxyethyl formate and 2-hydroxyethyl propanoate. These differences are attributed to the experimental conditions and to the ability of FTIR spectroscopy to directly monitor polar and low-volatility products in the gas phase. Such species are not efficiently sampled, retained or reliably quantified using standard GC-based techniques, particularly when SPME is employed, leading to their underrepresentation in GC–MS analyses.

Although 2-propoxyethanal and 2-ethyl-1,3-dioxolane were assigned in our experiments, they were not quantified.

For Cl-initiated reactions, the lower carbon recovery observed in the absence of NO suggests preferential formation of other nonquantified oxygenated products, such as ethoxypropanal and hydroperoxides, arising from RO_2_ + RO_2_ and RO_2_ + HO_2_ reaction pathways. In the presence of NO, RO_2_ chemistry is redirected toward alkoxy radical formation, leading to more volatile and readily detectable carbonyl products and a substantially higher carbon recovery. In NO_3_^•^ reactions, the formation of nitrated species dominates, resulting in low carbon recovery when only non-nitrated products are considered.

### Atmospheric Implications

The atmospheric impact of 2-PE has been evaluated through four key parameters: atmospheric lifetime (τ), Global Warming Potential (GWP), Photochemical Ozone Creation Potential (POCP), and the identity of the oxidation products. These calculations are commonly used to evaluate the environmental relevance of oxygenated VOCs (OVOCs) within air quality and atmospheric chemistry frameworks.

Atmospheric lifetimes were calculated using the expression τ_*R*_ = 1/*k*_*R*_[Ox], where *k*_*R*_ is the rate coefficient of the reaction and ([Ox]) is the typical atmospheric concentration of the oxidant: 1 × 10^6^ radical cm^–3^ (12 h average)^50^ for OH^•^, 5 × 10^8^ radical cm^–3^ ^51^ for NO_3_^•^, 1 × 10^3^ atoms cm^–3^ (average global concentration),^52^ or 1.3 × 10^5^ atoms cm^–3^ ^53^ (peak concentration in coastal and industrial areas) for Cl. Under these assumptions, the calculated lifetimes for 2-PE are 13 h (OH^•^), 77 days (NO_3_^•^), and 58 days (Cl, global mean conditions), decreasing to 10.7 h under peak Cl conditions.

Processes such as dry/wet deposition and heterogeneous reactions must also be considered, although the lack of data prevents quantifying the contributions of dry deposition and heterogeneous losses. Then, wet deposition lifetimes (τ_WD_) were estimated using Henry’s law constants (*k*_H_) reported by Sander (2023),^54^ applying the eq 6 proposed by Chen et al. (2003)^55^

6 $$\tau_{WD}=\frac{H_{atm}}{JRTk_{H}}$$

in eq 6, *H*_atm_ represents the mean atmospheric height (6 km), *J* the global average precipitation rate (100 cm/year), *R* the ideal gas constant (0.082 atm·L·mol^–1^·K^–1^), *T* the average tropospheric temperature (275 K). Values of *H*_atm_, *J*, and *T* were taken from Chen et al. (2003).^55^ *k*_H_ is the Henry’s law constant for 2-propoxyethanol (2-PE), 6.69 × 10^4^ mol L^–1^ atm^–1^, as reported by Sander (2023).^54^ The estimated lifetime obtained (τ_WD_) for 2-PE is 35 h.

The overall atmospheric lifetime considering all the homogeneous reactions with oxidants, is 9.51 h, which decreases to 7.47 h when wet deposition is included. According to these results, we can conclude that the reaction of 2-PE with OH radicals is the predominant atmospheric degradation process of this compound.

Because 2-PE is removed from the atmosphere on relatively short time scales, its atmospheric impact is governed primarily by the formation and fate of its oxidation products. Formaldehyde is a common intermediate formed during the atmospheric oxidation of many volatile organic (not specific to 2-PE) and it is classified as a potential carcinogen^56^ and, together with other low-molecular-weight aldehydes and organic nitrates, is a known contributor to urban photochemical pollution. The formation of propyl formate and other highly soluble esters (propionates, glycolates) suggests wet deposition processes or potential acid formation. Moreover, the multifunctional and highly oxygenated products generated exhibit low volatility, consistent with their potential to contribute to Secondary Organic Aerosol (SOA) formation, an outcome frequently associated with OVOC oxidation in both urban and regional atmospheres.^57^

To evaluate the 2-PE contribution to climate change, Global Warming Potential (GWP) was calculated based on atmospheric lifetime and IR absorption characteristics,^58^ using the approach of Hodnebrog et al. (2020).^59^ The resulting GWP values for 2-PE are 0.054, 0.013, and 0.0038 for time horizon of 20, 100, and 500 years, respectively. These values, all well below unity, confirm that 2-PE does not contribute meaningfully to climate forcing and that its atmospheric relevance is instead dominated by its reactive chemistry and secondary product formation.

The Photochemical Ozone Creation Potential (POCP) expresses the relative capacity of a VOC to form tropospheric ozone, comparing the ozone generated by its emission to that produced by an equivalent increase in a reference compound, typically ethene, using a photochemical model.^41^ For 2-PE, estimated POCP values are 47.4 under northwestern European conditions and 46.8 under urban U.S. conditions. Reported POCP values for related hydroxyethers fall within similar ranges.^8–10,19^ Compared to other organic compounds,^41^ hydroxy ethers show a moderate ozone forming potential, higher than that of alkanes, esters, and carboxylic acids, and comparable to ethers and alcohols, though lower than alkenes. This highlights that, despite its short atmospheric lifetime, 2-PE can contribute to local tropospheric ozone formation under VOC and NOx-rich conditions.

## Conclusions

This work provides the first experimental rate coefficients for the reactions of 2-propoxyethanol (2-PE) with Cl atoms and NO_3_ radicals together with a refine value for the reaction with OH^•^, measured under ambient temperature and pressure. The reactivity trend *k*_Cl_ > *k*_OH_ > *k*_NO3_ is consistent with established OVOC behavior and the resulting kinetic parameters are directly applicable to atmospheric mechanisms and chemical transport models. SAR schemes reproduce well the measured Cl and OH rate coefficients but underestimate NO_3_ reactivity, in agreement with observations for related hydroxyethers. This indicates that NO_3_ reactivity factors for multifunctional OVOCs would benefit from refinement to improve nighttime chemistry representation.

Combining FTIR and SPME/GC–MS results, the oxidation of 2-PE forms formaldehyde, acetaldehyde, and organic esters (mainly propyl formate, 2-hydroxyethyl formate, 2-hydroxyethyl propanoate). Under NO_*x*_ conditions, organic nitrates are also produced. Structure–Activity considerations and product measurements show that initial oxidation proceeds mainly by H-abstraction at the –CH_2_– group adjacent to the ether oxygen (routes II and III). A smaller contribution from route I is consistent with tentative evidence for 2-propoxyethanal.

Using representative oxidant levels, lifetimes indicate that reaction with OH^•^ is the predominant atmospheric sink of 2-PE in the troposphere; NO_3_^•^ contributes under polluted nighttime conditions, and Cl may be relevant in coastal/industrial air. Wet deposition, estimated from Henry’s law, can operate for soluble products, complementing gas-phase removal. The GWP of 2-PE is negligible, confirming that its atmospheric significance lies in reactive chemistry and secondary products, not in radiative forcing. The POCP (∼47) indicates a moderate ozone-forming potential, comparable to other hydroxyethers and higher than that of alkanes and many oxygenates, suggesting that 2-PE can contribute to local O_3_ formation under VOC- and NO_*x*_-rich conditions. The formation of multifunctional, polar, low-volatility products is consistent with a potential contribution to secondary organic aerosol (SOA).

Overall, 2-PE behaves as a short-lived OVOC whose atmospheric impact is governed by fast gas-phase oxidation (dominated by OH^•^) and formation of carbonyls, organic nitrates, and soluble ester type products. These findings provide the kinetic and mechanistic knowledge needed to evaluate the role of 2-PE in urban photochemistry, ozone formation, and the generation of secondary products within air-quality frameworks.

## Supplementary Material



## Notes

The Supporting Information includes kinetic plots for the reactions of 2-PE with OH^•^, NO_3_^•^, Cl, and Cl + NO radicals (Figures S1–S5), including FTIR-based time–concentration profiles and product evolution curves (Figures S3–S5). Chromatographic data showing the temporal evolution of reactants and products are provided for the OH^•^ and Cl + NO systems (Figures S6–S7). Tables S1–S3 compile SAR input parameters, chromatographic peak assignments, and EI mass spectra of 2-PE and its products. Table S4 presents the estimated percentages of attack at each position of the 2-PE molecule obtained using SAR methodology, while Table S5 includes additional estimations based on the Jenkins approach. Detailed descriptions of the SAR calculation procedure are also provided. The correction methodology for product concentrations based on the formalism of Tuazon et al. (1986),^29^ accounting for secondary reactions with oxidants under the experimental conditions is developed in the Supporting Information. The detailed explanation of weighted average calculations of the rate coefficients is also included in the Supporting Information (PDF)
