# Supplementary material for: Atmospheric Reactivity and Environmental Implications of 2‑Propoxyethanol: Kinetic and Mechanistic Insights into Its Oxidation by OH, NO3, and Cl
Source: ACS Environ Au. 2026 Jun 18;6(5):829–40. doi: 10.1021/acsenvironau.6c00061 (PMC13602334; doi:10.1021/acsenvironau.6c00061)
Supplement: Supplementary file 1 [file vg6c00061_si_001.pdf]

Supporting Information

Atmospheric Reactivity and Environmental Implications of 2-Propoxyethanol: Kinetic and Mechanistic Insights into Its Oxidation by OH, NO_3, and Cl

Sagrario Salgado[1,2], Inmaculada Aranda[1,2], Pilar Martín[1,2], Florentina Villanueva[2,3], Elena Molina[1,2], Beatriz Cabañas[1,2]*

AUTHOR ADDRESS (Word Style "BC_Author_Address"). [1]Universidad de Castilla-La Mancha, Departamento de Química Física, Facultad de Ciencias y Tecnologías Químicas, Avda. Camilo José Cela s/n 13071, Ciudad Real, Spain. [2]Universidad de Castilla-La Mancha, Instituto de Combustión y Contaminación Atmosférica (ICCA), Camino de los Moledores s/n, 13071, Ciudad Real, Spain. [3] Universidad de Castilla-La Mancha, Departamento de Química Física, Escuela de Ingeniería Industrial y Aeroespacial, Avenida Carlos III, s/n, 45003, Toledo Spain.

* Corresponding autor: Sagrario.salgado@uclm.es

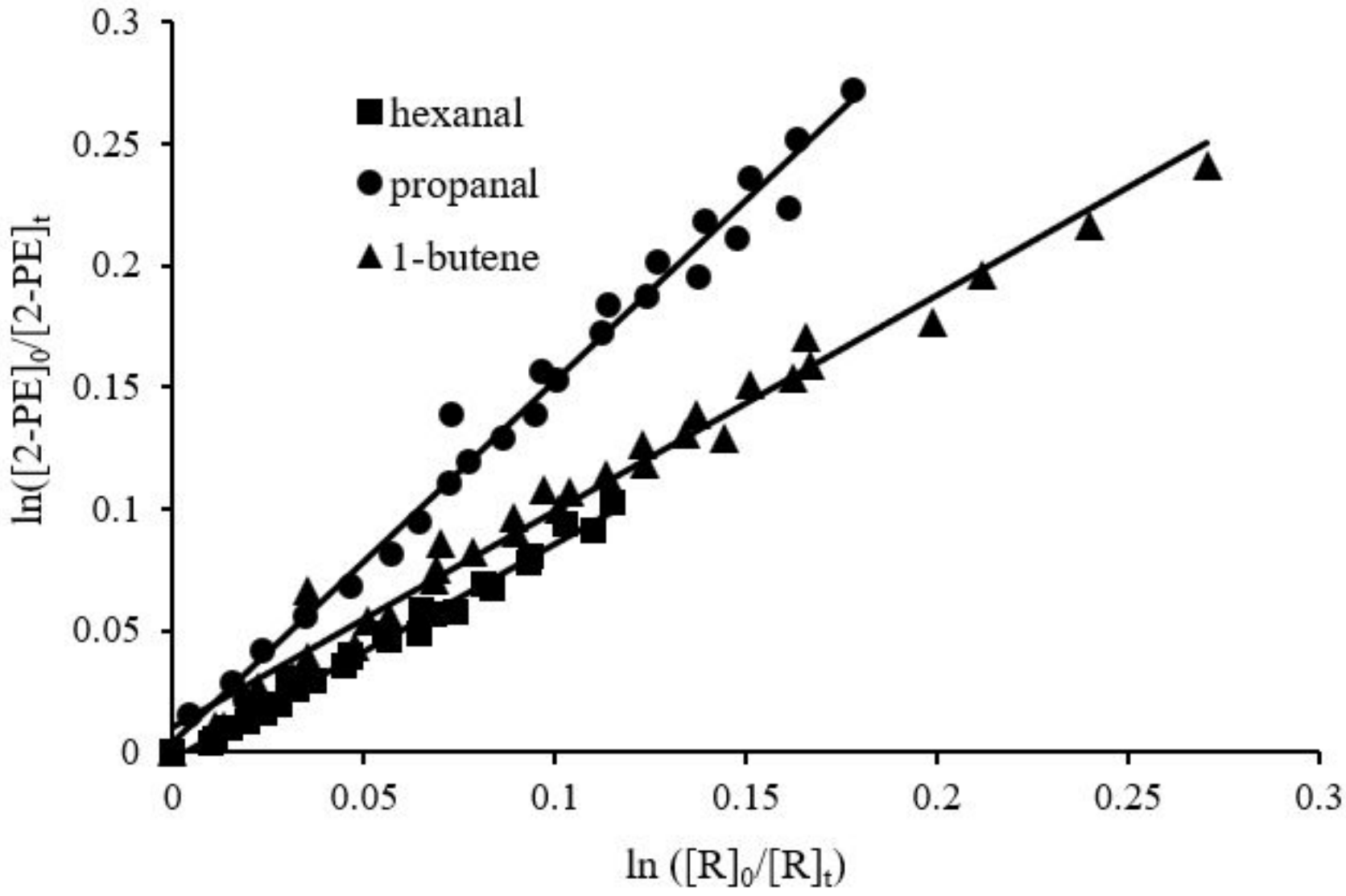

Figure S1. Plot according to equation 1 for the reaction of 2-PE with OH radicals with three reference compounds.

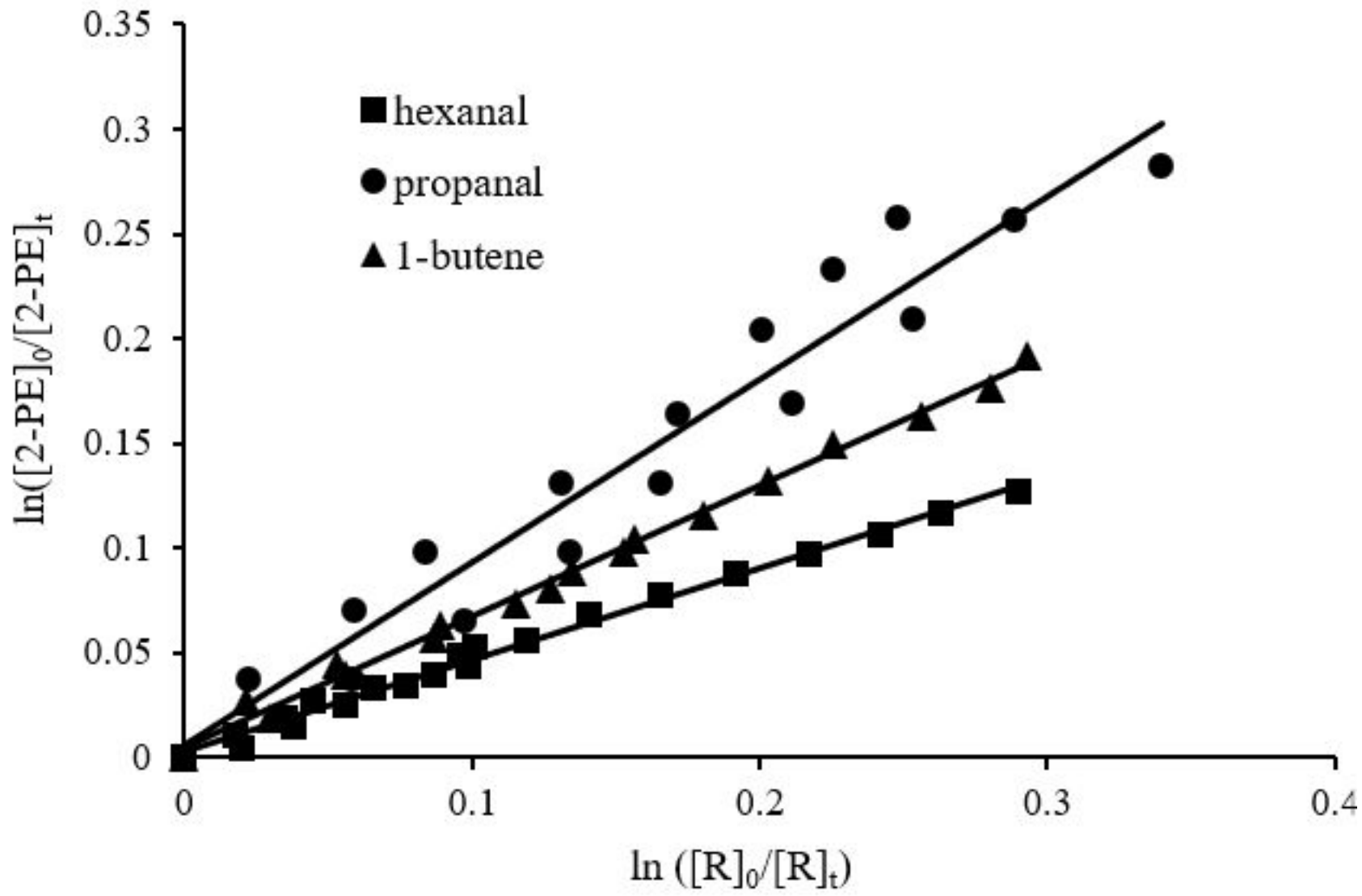

Figure S2. Plot according to equation 1 for the reaction of 2-PE with NO_3 radicals with three reference compounds.

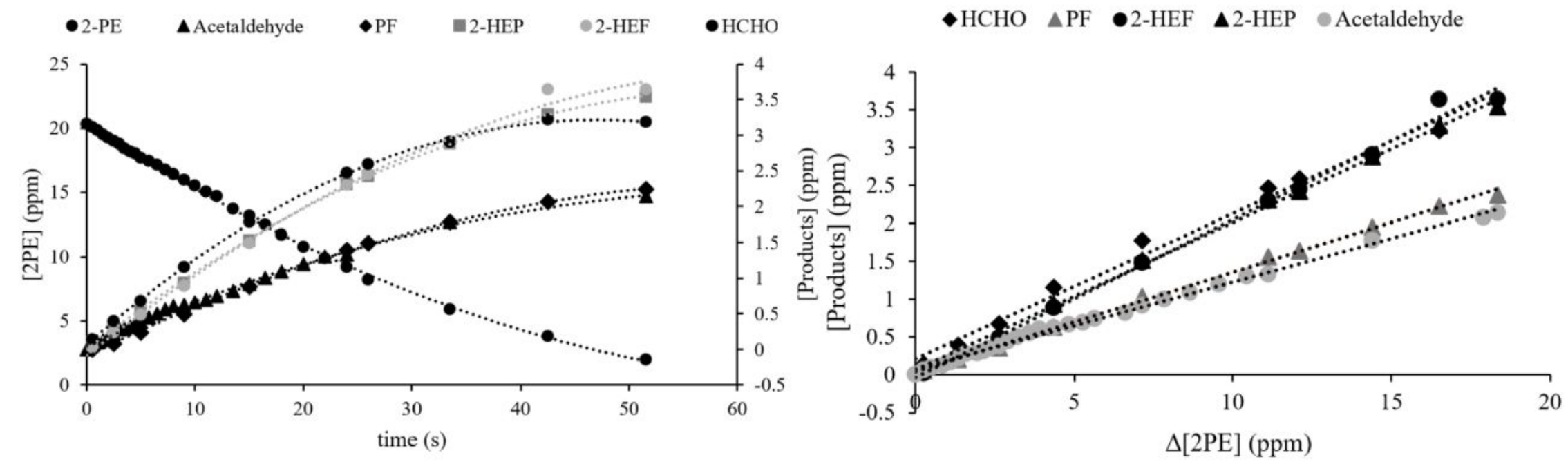

a) b)

Figure S3. For Cl reactions with 2-PE: a) Time-concentration profiles of reactant (2-PE) and products as measured by FTIR. b) Plots of the variation of the concentration of the quantified products as a function of the reactant variation. [PF]= propyl formate; [2-HEP]= 2-hydroxy ethyl propanoate; [2-HEF]= 2-hydroxy ethyl formate.

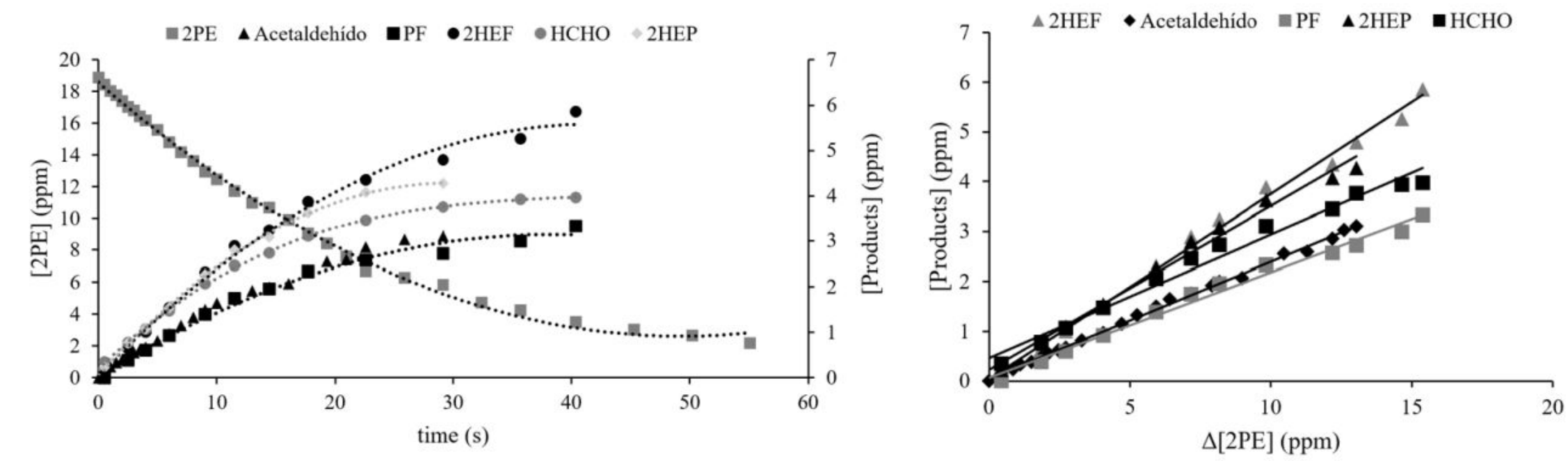

a) b)

Figure S4. For Cl+NO reactions with 2-PE: a) Time-concentration profiles of reactant (2-PE) and products as measured by FTIR. b) Plots of the variation of the concentration of the quantified products as a function of the reactant variation. [PF]= propyl formate; [2-HEP]= 2-hydroxy ethyl propanoate; [2-HEF]= 2-hydroxy ethyl formate.

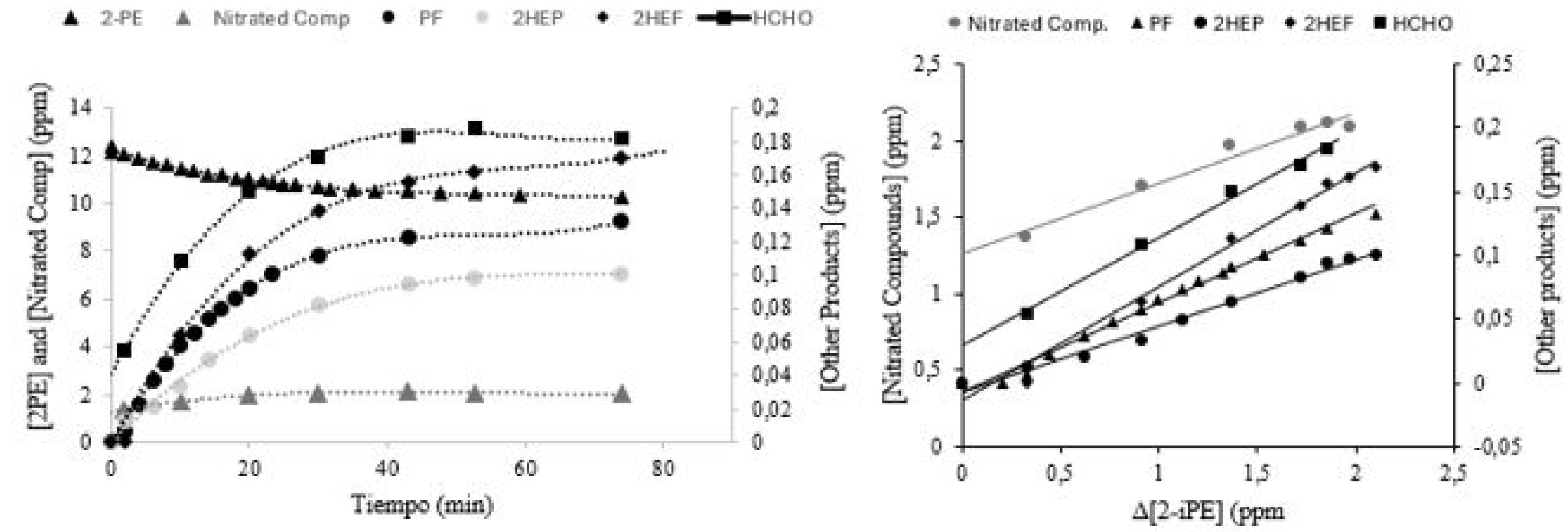

a) b)

Figure S5. $NO_3\cdot$ reactions with 2-PE: a) Time-concentration profiles of reactant (2-PE) and products as measured by FTIR. b) Plots of the variation of the concentration of the quantified products as a function of the reactant variation. [PF]= propyl formate; [2-HEP]= 2-hydroxy ethyl propanoate; [2-HEF]= 2-hydroxy ethyl formate.

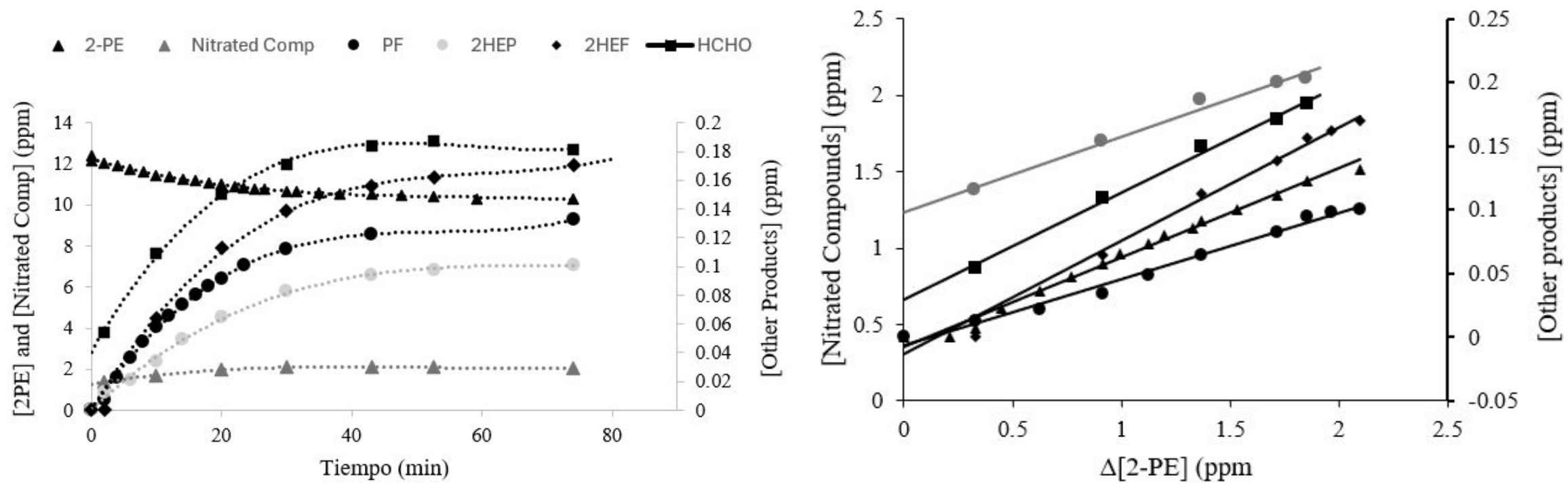

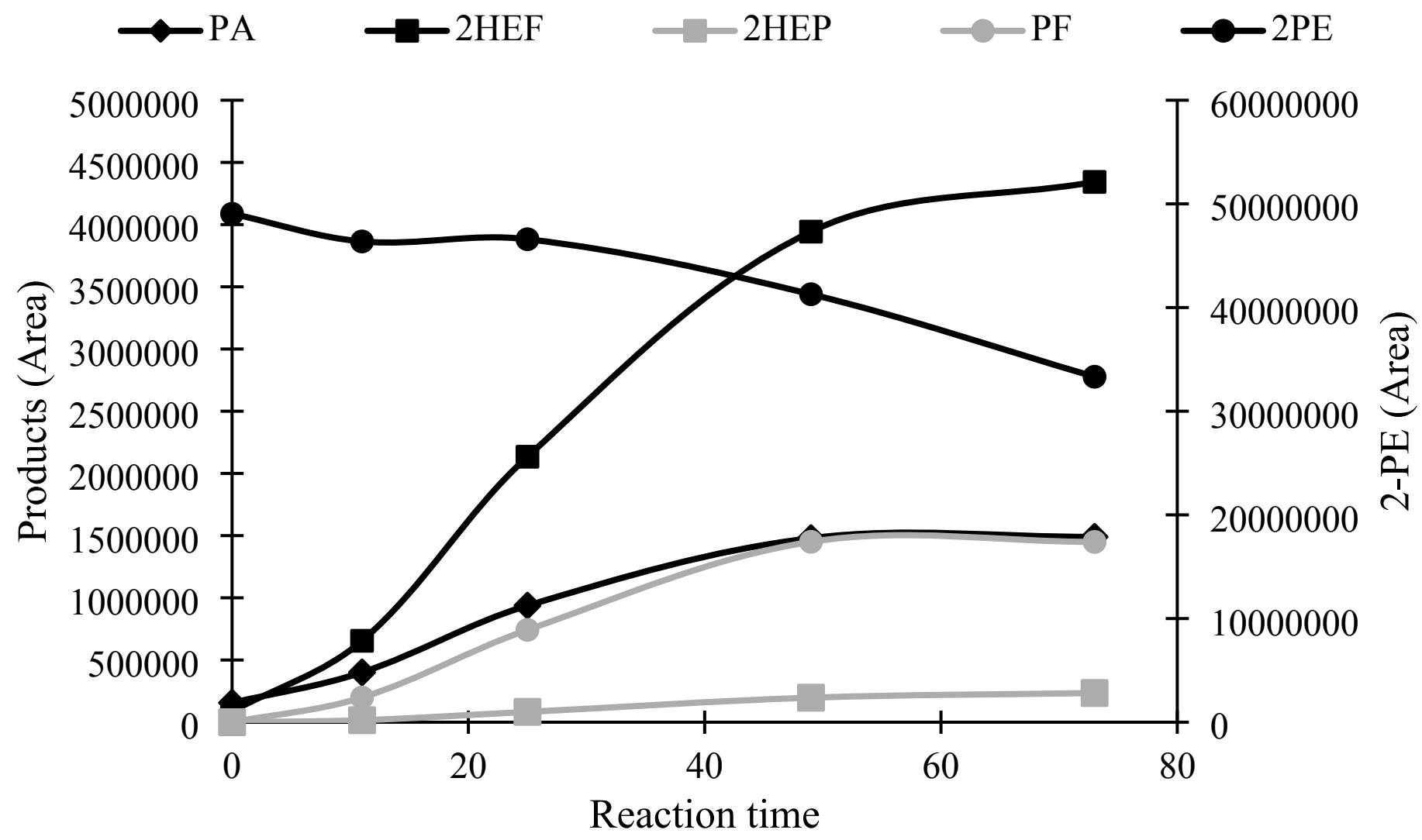

Figure S6. Plot of chromatographic areas of 2-PE and reaction products, as a function of the reaction time, for the 2-PE+·OH· reaction. [PF]= propyl formate; [2-HEP]= 2-hydroxy ethyl propanoate; [2-HEF]= 2-hydroxy ethyl formate; [PA]=2-propoxyethanal.

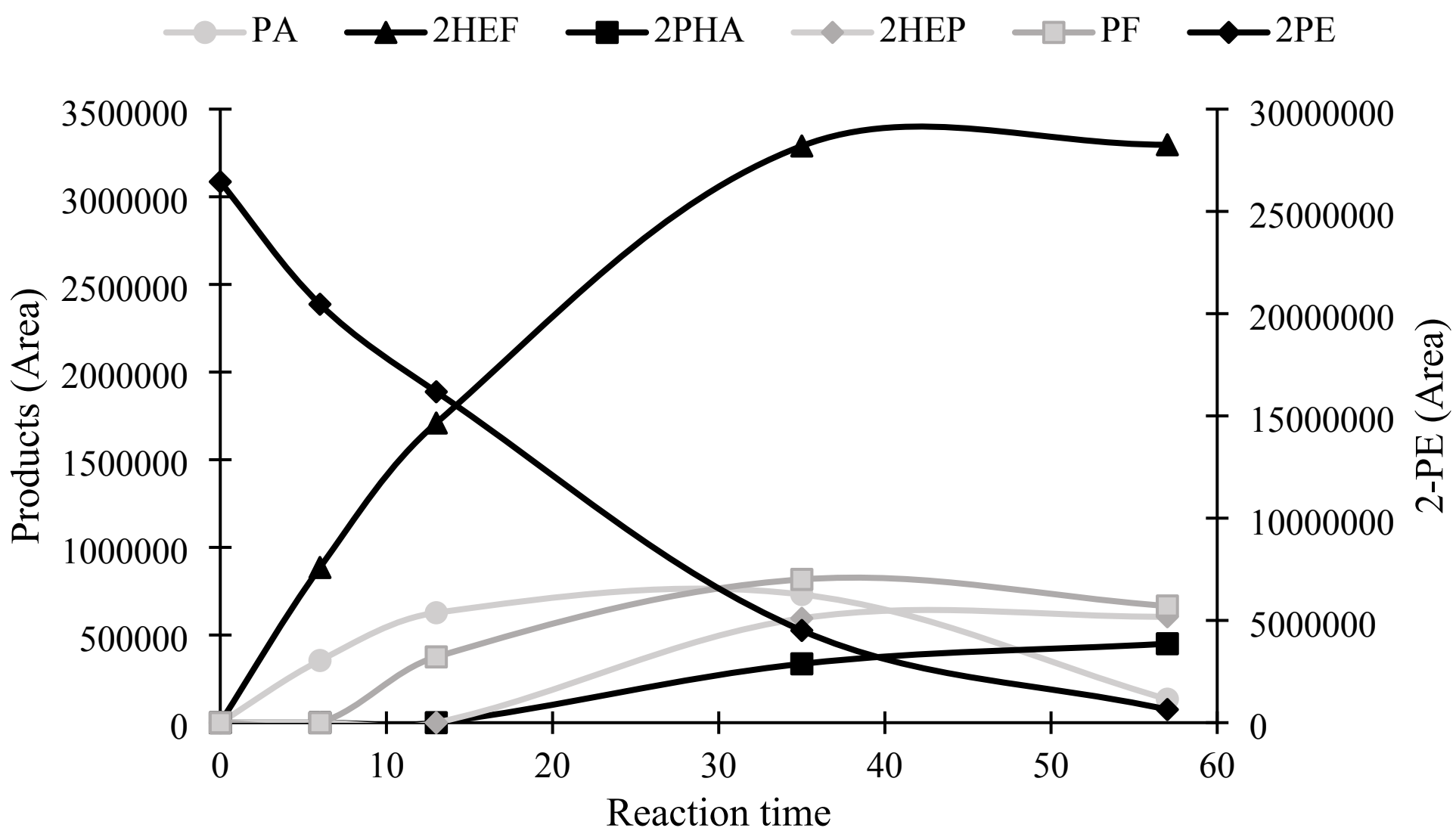

Figure S7. Plot of chromatographic areas of 2-PE and reaction products, as a function of he reaction time, for the 2PE + Cl + NO reaction. [PF]= propyl formate; [2-HEP]= 2-hydroxy ethyl propanoate; [2-HEF]= 2-hydroxy ethyl formate; [PA]=2-propoxyethanal; [2PHA]=propyl hydroxyacetate.

Table S1. The values used for SARs calculations, of k_{prim}, k_{sec}, k_{terc} and k_{OH}, as well as the reactivity factors (F(R) for functional groups.

**Constant (cm^3 $molec^{-1}s^{-1}$)**	**Cl/10^{-11}**	**OH/10^{-13}·**	**NO_3·/10^{-17}**
k_{prim}	2.84	1.36	10
k_{sec}	8.98	9.34	2.56
k_{tecr}	6.48	1.94	0.105
k_{OH}		1.40	2
**Factors**	**Cl**	**OH·**	**NO_3·**
F(-CH_3-	1	1	1
F(-CH_2-; -CH<;>C<)	0.8	1.23	1.02 (-CH_2-)<br>1.61 (-CH<)<br>2.03 (>C<)
F(-OH)	1.18	2.35	18
F(-O)	1.45	4.13	58 (-O-CH_2-)<br>23 (-O-CH<)
F(-CR_2-O-R)	0.4	2.84	10 (>CH-O-R)<br>1 (-CH_2-O-R)

Table S2. Summary of chromatographic peaks and assignation:

t_R	compound	OH·	Cl	Cl+NO	NO_3
7.28	2-PE	reactant	reactant	reactant	reactant
7.84	2-hydroxyethylformate (2HEF)	Yes	Yes	Yes	Yes
6.74	¿ propoxyetanal?	Yes	Yes	Yes	Yes
10.20	2-hydroxyethylpropanoate (2HEP)	Yes	Yes	Yes	Yes
3.61	Propyl formate (PF)	Yes	Yes	Yes	Yes
8.41	2-hydroxyacetate propyl (2PHA)		Yes	Yes	
10.33	¿nitrated compound?				Yes
8.06	unidentified	Yes	No		
6.09	2-ethyl-1,3dioxolane (2E1,3D)	Yes	No	Yes	No

Table 3S. Mass spectra of 2-PE and the reaction products, obtained with an EI ionization source. Possible assignment and fragmentation.

t_R (min)	MS spectrum (EI)	Assignment and fragmentation of products
2PE<br>7.27	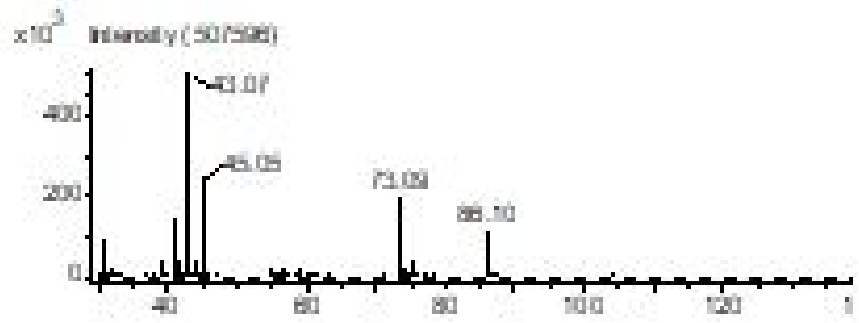	2-propoxy ethanol<br>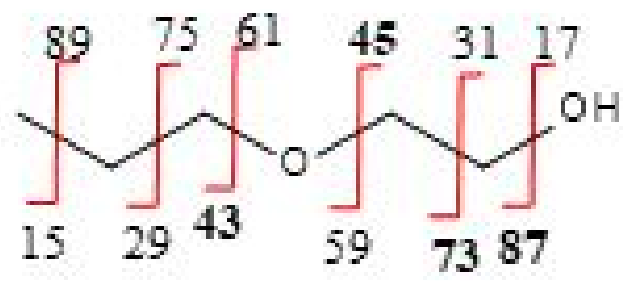
7.84	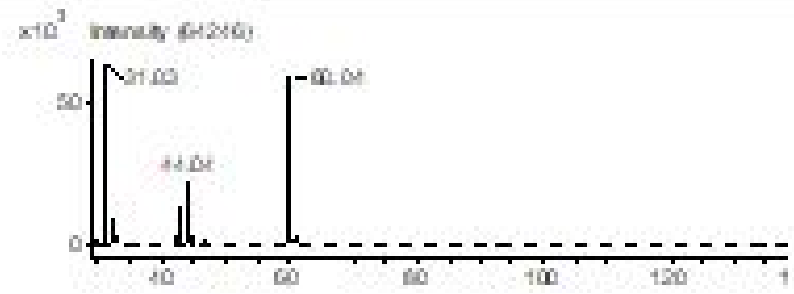	2-hydroxyethyl formate<br>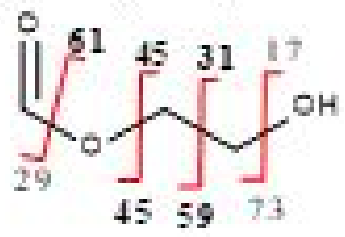
6.74	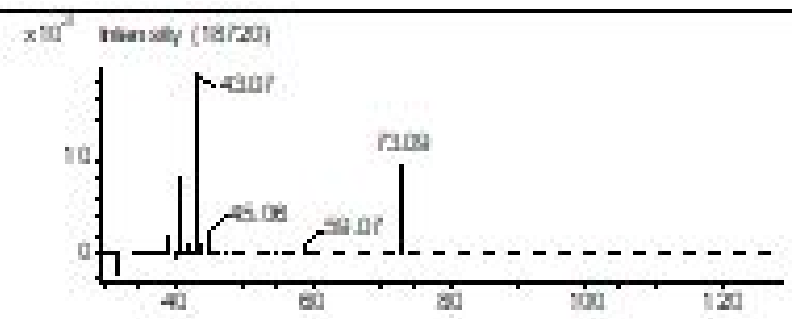	
10.21	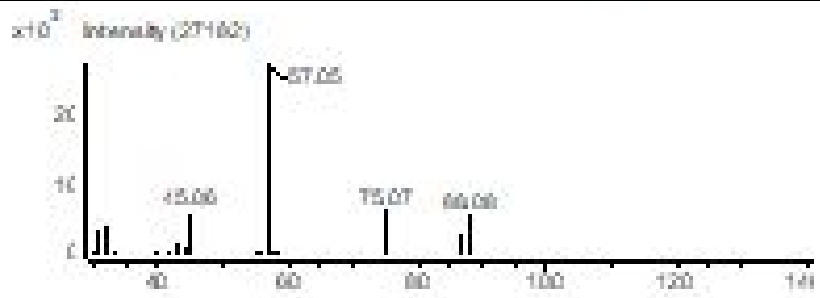	2-hydroxyethyl propanoate<br>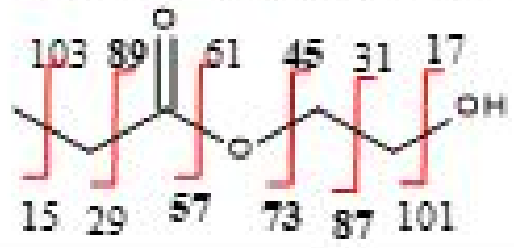
3.6	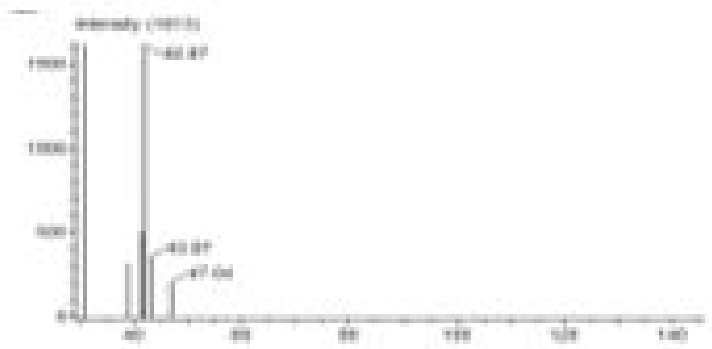	Propyl formate<br>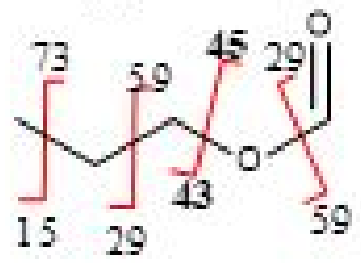
8.42	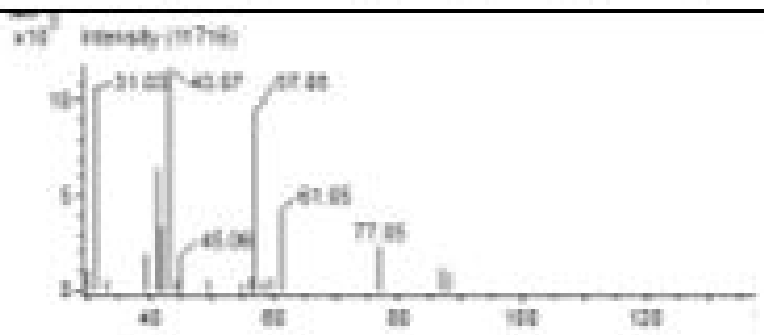	2-hydroxyacetate propyl<br>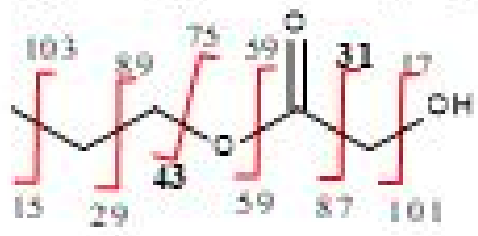 Low percentage of similitude(~30%)

Weighted average calculations

First, the rate coefficient k_S is obtained from the slope (*pte*) of the relative rate plot using the reference rate coefficient k_R. The explanation and the used equation are detailed in the manuscript:

$$\sigma(k_S) = \sqrt{(k_R \cdot \sigma_{\text{pte}})^2 + (\text{pte} \cdot \sigma_{k_R})^2}$$

For each compound, at least three different reference compounds are employed, and each experiment is performed in triplicate. Consequently, several independent values of k_S are obtained, which are combined using weighted statistics to account for their individual uncertainties. The weighted average $\grave{x}$ is calculated as:

$$\grave{x} = \frac{\sum_{i=1}^{N} w_i\, x_i}{\sum_{i=1}^{N} w_i}$$

where x_i are the individual rate coefficients, N is the number of determinations, and the weights w_i are defined as the inverse of the variance:

$$w_i = \frac{1}{\sigma_i^2}$$

The uncertainty associated with the weighted mean is calculated as:

$$\sigma = \sqrt{\frac{1}{\sum_{i=1}^{N} w_i}}$$

This approach ensures that determinations with smaller uncertainties contribute more strongly to the final value, while fully accounting for the experimental variability and reference rate uncertainties.

SAR Calculations

To estimate the rate coefficients of the reactions of 2-PE with OH·, NO_3· and Cl, Structure-Activity Relationships (SAR) have been used. The methodology is based on the database of experimental kinetics coefficients available to estimate the reactivity factors (F(R); R = functional group) for each functional group depending on the type of carbon to which it is attached (primary, secondary, tertiary or quaternary). In this work, the factors proposed by Calvert et al., 2011 have been used for Cl reactions and OH radical reactions and the proposed by Kerdouci et al., 2014 for NO_3 radical reaction.

2PE does not have double bonds in its structure, so the reaction will proceed by abstraction by the oxidant of the hydrogen atoms of the molecule. The global abstraction rate coefficients have been calculated using the values of k_{prim}, k_{sec}, k_{terc} y k_{OH}, and the reactivity factors (F(R) for functional groups presented in table S1, and using the following equation: (equation 3).

$$k_{abs} = \sum k_{prim}F(X) + \sum k_{sec}F(X)F(Y) + \sum k_{tert}F(X)F(Y)F(Z) + \sum k_{OH} \qquad (S.1)$$

There are six potential pathways. We can calculate the approximate percentage of attacks at each position for the reaction with the three oxidants, as shown below.

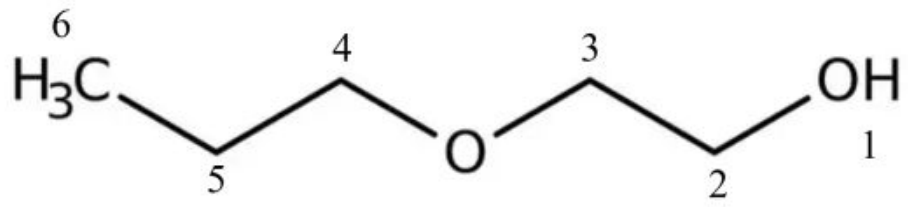

Chlorine Atoms

k_1= -

k_2= $8.95\times10^{-11}\times$F(-CR2OR)$\times$F(-OH)= $8.95\times10^{-11}\times0.4\times1.18$= 4.22×10^{-11} $cm^3molecule^{-1}\ s^{-1}$.

k_3= $8.95\times10^{-11}\times$F(-O-)$\times$F(-CR2OR-)= $8.95\times10^{-11}\times1.45\times0.4$= 5.19×10^{-11} $cm^3molecule^{-1}\ s^{-1}$.

k_4= $8.95\times10^{-11}\times$F(-O-)$\times$F(-CH2-)= $8.95\times10^{-11}\times1.45\times0.8$= 1.04×10^{-10} $cm^3molecule^{-1}\ s^{-1}$.

k_5= $8.95\times10^{-11}\times$F(-CR2OR-)$\times$F(-CH3)= $8.95\times10^{-11}\times0.4\times1$= 3.58×10^{-11} $cm^3molecule^{-1}\ s^{-1}$.

k_6= $2.84\times10^{-11}\times$F(-CH2-)= $2.84\times10^{-11}\times0,8$= 2.27×10^{-11} $cm^3molecule^{-1}\ s^{-1}$.

k_{Cl} =2.56×10^{-10} $cm^3molecule^{-1}\ s^{-1}$

R=H or alkyl group.

OH radicals

k_1= 1.7×10^{-14}cm^{3}molecule^{-1} s^{-1}.

k_2= 9.34×10^{-13}×F(-CH2OR-)×F(-OH)= 9.34×10^{-13}×2.84×2,35=6.23×10^{-12} cm^{3}molecule^{-1} s^{-1}.

k_3= 9.34×10^{-13}×F(-CH2OH-)×F(-O-R)=9.34×10^{-13}×2.84×4,13=1.096×10^{-11} cm^{3}molecule^{-1}s^{-1}.

k_4= 9.34×10^{-13}×F(-CH2-)×F(-O-R)=9.34×10^{-13}×1.23×4.13=4.75×10^{-12} cm^{3}molecule^{-1}s^{-1}.

k_5= 9.34×10^{-13}×F(-CH2OR-)×F(-CH3)=9.34×10^{-13}×2.84×1=2.65×10^{-11} cm^{3}molecule^{-1}s^{-1}.

k_6= 1.36×10^{-13}×F(-CH2-)= 1.36×10^{-13}×1.23= 1.67×10^{-13} cm^{3}molecule^{-1} s^{-1}.

k_{OH}=2.49×10^{-11} cm^{3}molecule^{-1} s^{-1}

NO_3 radicals

k_1= 2×10^{-17}cm^{3}molecule^{-1} s^{-1}.

k_2= 2.56×10^{-17}×F(-CH2OR)×F(-OH)= 2.56×10^{-17}×1×18= 4.61×10^{-16} cm^{3}molecule^{-1} s^{-1}.

k_3= 2.56×10^{-17}×F(-CH2OH-)×F(-OCH2-)= 2.56×10^{-17}×1×58= 1.48×10^{-15} cm^{3}molecule^{-1} s^{-1}.

k_4= 2.56×10^{-17}×F(-OCH2-)×F(-CH2-)= 2.56×10^{-17}×58×1.02= 1.51×10^{-15} cm^{3}molecule^{-1} s^{-1}.

k_5= 2.56×10^{-17}×F(-CH2OR-)×F(-CH3)= 2.56×10^{-17}×1×1= 2.56×10^{-17} cm^{3}molecule^{-1} s^{-1}.

k_6= 1×10^{-18}×F(-CH2-)= 1×10^{-18}×1.02= 1.02×10^{-18} cm^{3}molecule^{-1} s^{-1}.

k_{NO3}=3.51×10^{-15} cm^{3}molecule^{-1} s^{-1}

R=H o alkyl group

Table S4. Estimation of attack percentages at each 2-PE position using the SAR method.

Attack percentage (%)	Position 1 (-OH)	Position 2 (-CH_2-OH)	Position 3 (-O-CH_2-)	Position 4 (-CH_2-O-)	Position 5 (-CH_2-)	Position 6 (-CH_3)
**Cl atoms**		16.5	20.2	40.5	14	8.9
**OH radicals**	0.6	25	44	19	10.7	0.7
**NO_3 radicals**	0.6	13.1	42.3	43.2	0.73	0.03

Jenkins Calculations

OH radicals

k_1= 1.4×10^{-13} cm^{3}molecule^{-1} s^{-1}.

k_2= 7,69×10^{-13}×F(-CH2OR-)×F(-OH)= 7,69×10^{-13}×3.5×3.6=9.69×10^{-12} cm^{3}molecule^{-1} s^{-1}.

k_3= 7,69×10^{-13}×F(-CH2OH-)×F(-O-R)= 7,69×10^{-13}×1.8×3.6=4.98×10^{-12} cm^{3}molecule^{-1}s^{-1}.

k_4= 7,69×10^{-13}×F(-CH2-)×F(-O-R)= 7,69×10^{-13}×1.35×3.6=3.74×10^{-12} cm^{3}molecule^{-1}s^{-1}.

k_5= 7,69×10^{-13}×F(-CH2OR-)×F(-CH3)= 7,69×10^{-13}×3.5×1=2.69×10^{-12} cm^{3}molecule^{-1}s^{-1}.

k_6= 1.30×10^{-13}×F(-CH2-)= 1.30×10^{-13}×1.35= 1.76×10^{-13} cm^{3}molecule^{-1} s^{-1}.

k_{OH}=2.14×10^{-11} cm^{3}molecule^{-1} s^{-1}

Table S5. Estimation of attack percentages at each 2-PE position using the SAR method.

**Attack percentage (%)**	**Position 1 (-OH)**	**Position 2 (-CH_2-OH)**	**Position 3 (-O-CH_2-)**	**Position 4 (-CH_2-O-)**	**Position 5 (-CH_2-)**	**Position 6 (-CH_3)**
**OH radicals**	0.65	45.28	23.27	17.48	12.57	0.82

The rate coefficient for the gas-phase reaction of 2-propoxyethanol with OH radicals was also estimated using the Structure-Activity Relationship (SAR) method originally developed by Jenkins et al. and subsequently refined for oxygenated volatile organic compounds. The total rate coefficient for reaction with OH is obtained by summing the contributions from H-atom abstraction at all distinct carbon-bound hydrogen sites in the molecule. Each abstraction site is characterized according to its local chemical environment, considering the type of carbon atom (primary, secondary or tertiary), its substitution pattern, and the influence of neighboring functional groups.

The total rate coefficient for reaction with OH radicals is given by:

$k_{\mathrm{OH}} = \sum_i k_i$ where k_i is the site-specific abstraction rate coefficient for hydrogen abstraction at carbon site i.

Site-specific abstraction rate coefficient

For each abstraction site i, the contribution k_i was calculated as:

$k_i = N_i \cdot k_{\text{base},i} \cdot \prod_j F_{j,i}$ where: N_i is the number of equivalent hydrogen atoms at site i, $k_{\text{base},i}$ is the base rate constant for abstraction from a carbon atom of the corresponding type (primary or secondary C–H), $F_{j,i}$ are multiplicative correction factors accounting for substituent effects from neighboring functional groups.

Base rate constants and correction factors were taken from the parameterization provided by Jenkins et al. for oxygenated hydrocarbons. For each abstraction site, the base rate constant was modified using correction factors to account for the influence of the ether oxygen atom, the hydroxyl group and substitution at adjacent carbon atoms. Thus, for a given abstraction site *i*, the effective abstraction rate was calculated as:

$k_i = N_i \cdot k_{\text{base}}(\text{C–H type}) \cdot F_{\text{O},i} \cdot F_{\text{OH},i} \cdot F_{\text{sub},i}$ where: $F_{\text{O},i}$ corrects for proximity to the ether oxygen, $F_{\text{OH},i}$ accounts for the presence of the hydroxyl group, $F_{\text{sub},i}$ accounts for alkyl substitution. All correction factors were applied exactly as specified in the Jenkins SAR scheme for multifunctional oxygenated compounds.The overall OH rate coefficient for 2-propoxyethanol was obtained by summing the individual contributions from all abstraction sites:

$$k_{\text{OH}} = k_2 + k_3 + k_4 + k_5$$

This summation yielded the SAR-predicted bimolecular rate coefficient reported in the manuscript.

Calculation of branching fractions

The fractional contribution of each abstraction site to the total rate coefficient was calculated as:

$$f_i = \frac{k_i}{k_{\text{OH}}}$$

These fractions were used to estimate the relative importance of the four initial abstraction pathways (routes I–IV) shown in Scheme 1 and reported in Table S4. The branching ratios are provided to support qualitative mechanistic discussions and are not intended as exact experimental values

Formalism of Tuazon et al. (1986).

The correction procedure described below is applied considering only chemical loss of reaction products due to their reaction with the oxidant and neglecting possible photolysis processes, which were found to be negligible under the experimental conditions of this work:

2-PE + Ox → ∑ Y product1 ($k_{2\text{-PE}}$) (SI)

Product1 + Ox → Products2 (k_p) (SII)

Where Y is the yield of the primary product (product1) from the oxidation of 2-PE.

If we assume that the concentration of oxidant was constant during the irradiation period, then:

$$[2-\mathrm{PE}]_{t_2} = [2-\mathrm{PE}]_{t_1} \cdot e^{-\left(k_{2-PE} \cdot [\mathrm{Ox}]\right)(t_2 - t_1)} \qquad [\mathrm{S2}]$$

From Equation [S2] it is possible to calculate the concentration of oxidant in the system.

Using the relationship obtained by Tuazon et al. (1986) the corrected concentration of the reaction products can be calculated as follows:

$$[\mathrm{product1}]_{t_2} = [\mathrm{product1}]_{t_1} \cdot e^{-\left(k_p \cdot [\mathrm{Ox}]\right)(t_2 - t_1)} + \frac{Y_{t_1-t_2} \cdot [2-\mathrm{PE}]_{t_1} \cdot k_S[Ox]}{\{(k_P - k_S) \cdot [Ox]\}} \cdot \left[e^{-(k_S \cdot [Ox]) \cdot (t_2 - t_1)} - e^{-(k_P[Ox]) \cdot (t_2 - t_1)}\right] \qquad [\mathrm{S3}]$$

Where $[\text{2-PE}]_{t_1}$, $[\text{product1}]_{t_1}$, and $[\text{2-PE}]_{t_2}$, $[\text{product1}]_{t_2}$ are the observed concentrations of the 2-PE and the products at times t_1 and t_2 respectively, and $Y_{t_1-t_2}$ is the yield of formation of the individual products on the period of time (t_1 - t_2).

By means of equations [S2] and [S3], $Y_{t1\text{-}t2}$ can be calculated. The concentration of the reaction products, corrected for the reaction with radical is given by:

$$[product1]_{t_2}^{corr} = [product1]_{t_1}^{corr} + Y_{t_1-t_2} \cdot \left([2-\mathrm{PE}]_{t_1} - [2-\mathrm{PE}]_{t_2}\right) \qquad [\mathrm{S4}]$$

Where $[\text{product1}]_{t_1}^{\text{corr}}$ and $[\text{product1}]_{t_2}^{\text{corr}}$ are the concentrations of the product1 corrected at times t_1 and t_2 respectively:

The value of $k_{2\text{-PE}}$ has been obtained in this work. The value of k_P is taken from the bibliography.
